# Laser Synthesis in Liquid Induced Lattice Distortion in PtFeSn/Activated Carbon for Enhanced Methylcyclohexane Dehydrogenation

**DOI:** 10.1002/smtd.202500474

**Published:** 2025-06-23

**Authors:** Zheng Wang, Hossein Akhoundzadeh, Mudi Wu, Mingwu Tan, Yizhong Huang, Rong Xu

**Affiliations:** ^1^ School of Chemistry Chemical Engineering and Biotechnology Nanyang Technological University 62 Nanyang Drive Singapore 637459 Singapore; ^2^ School of Materials Science and Engineering Nanyang Technological University 50 Nanyang Avenue Singapore 639798 Singapore; ^3^ Institute of Sustainability for Chemicals Energy and Environment (ISCE2) Agency for Science Technology and Research (A*STAR) 1 Pesek Road, Jurong Singapore 627833 Singapore

**Keywords:** laser synthesis, lattice distortion, MCH dehydrogenation

## Abstract

Methylcyclohexane (MCH) has emerged as one of the most promising liquid organic hydrogen carriers (LOHCs) for H_2_ storage and long‐distance transportation. Developing efficient, selective, and stable catalysts for MCH dehydrogenation is essential to make the process viable for practical applications. In this study, a platinum‐iron‐tin alloy supported on activated carbon (PtFeSn/AC) is reported, prepared via laser synthesis in liquid (LSL), exhibiting excellent dehydrogenation performance. The rapid crystallization and quenching inherent to the LSL process kinetically trap lattice distortions in the PtFeSn/AC catalyst due to atomic radius mismatches among Pt, Fe, and Sn. These distortions generate strain effects that create a local unsaturated coordination environment and downshift the *d*‐band center of the catalyst, thereby enhancing the exposure of active sites and facilitating the desorption of toluene (TOL). As a result, the PtFeSn/AC catalyst demonstrates exceptional dehydrogenation performance, achieving a hydrogen evolution rate of 2625 mmol g_Pt_^−1^ min^−1^ under a weight hourly space velocity (WHSV) of 27.7 h^−1^. Notably, the catalyst exhibits remarkable stability, with only a 3.2% drop in conversion after 193 h of continuous reaction. Additionally, TOL selectivity remains extraordinarily high at 99.96%. This work provides critical insights into the design of high‐performance catalysts via non‐conventional synthesis methods for practical applications.

## Graphical abstract

A Pt‐Fe‐Sn alloy catalyst supported on activated carbon is synthesized via laser synthesis in liquid, introducing lattice distortions that enhance active site exposure and toluene desorption. The catalyst achieves a hydrogen evolution rate of 2625 mmol gPt⁻¹ min⁻¹ with 99.96% selectivity and excellent stability, offering a promising strategy for efficient MCH dehydrogenation in LOHC systems.

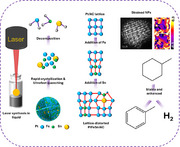

## 1 Introduction

The global distribution of renewable energy sources significantly differs from the geographical distribution of energy demand. Thus, energy transportation across the regions using hydrogen generated from renewables holds great promise for defossilization. Due to the low volumetric energy density of hydrogen at ambient conditions, various methods, such as liquefied hydrogen, compressed hydrogen gas pipelines, and metal hydrides, have been explored as potential solutions.^[^ ^1^, ^2^ ^]^ Nevertheless, these approaches still face significant challenges, including high infrastructure costs, low storage capacities or limited lifespans.^[^ ^3^ ^]^ As a result, developing a cost‐effective technology for hydrogen storage and transportation remains a key challenge for the widespread adoption of hydrogen as a clean energy source.

LOHCs offer a solution by chemically bonding hydrogen to liquid organic compounds, enabling hydrogen to be handled and transported in a liquid form.^[^ ^4^ ^]^ MCH represents one of the most promising LOHCs due to its low toxicity, moderate hydrogen storage capacity (6.1 wt%, 1.55 MWh m^−3^), and ability to be stored and transported using the existing petroleum infrastructure.^[^ ^5^, ^6^ ^]^ However, the efficiency of the dehydrogenation reaction of MCH needs to be further improved to make viable commercial applications. Conventional Pt‐based catalysts are often limited by their high cost and unsatisfactory stability.^[^ ^7^ ^]^ To achieve efficient and durable MCH dehydrogenation, strategies such as controlling Pt cluster sizes or alloying Pt with other metals (e.g., Fe, Zn, and Mn) have been proven effective in reducing Pt loading and minimizing coke formation.^[^ ^8^, ^9^, ^10^, ^11^, ^12^, ^13^, ^14^, ^15^, ^16^, ^17^ ^]^ However, challenges still remain in achieving efficient desorption of coke precursors to ensure long‐term durability under a pure MCH feed without co‐feeding hydrogen or inert carrier gases.^[^ ^18^ ^]^

Lattice defects of catalyst nanoparticles have been reported to be effective in modulating the interaction between catalysts and reaction molecules and significantly enhancing the catalytic properties.^[^ ^19^, ^20^ ^]^ By alloying Pt with other metals, atomic‐level strain can be induced due to differences in atomic radii among these elements.^[^ ^21^ ^]^ These strain effects alter the electronic structure of the alloy. Thus, the adsorption energy of reactive species can be tuned by the varied coordination environment of the surface sites in a few reactions such as oxygen reduction reaction and hydrogen evolution reaction.^[^ ^22^, ^23^, ^24^ ^]^ Nonetheless, this effect is much less explored in designing more efficient catalysts for high‐temperature MCH dehydrogenation.

In this study, inspired by the laser scanning ablation method reported earlier,^[^ ^25^ ^]^ we synthesized a Pt‐Fe‐Sn alloy supported on activated carbon (AC) using a modified laser synthesis in liquid (LSL) method. Substantial lattice distortion and strain can be incorporated into the resultant PtFeSn alloy nanoparticles due to the ultrafast quenching from high temperatures during the synthesis. This led to a decrease in the coordination number of Pt and Fe, the enhanced overlap of *d* band and a consequent downshift of the *d*‐band center, as revealed by spectroscopic methods. Correspondingly, the strained catalyst exhibits easier desorption of TOL, which helps suppress the coke formation. The optimal PtFeSn/AC catalyst exhibited a high H_2_ evolution rate of 2625 mmol g_Pt_^−1^ min^−1^ at a weight hourly space velocity (WHSV) of 27.7 h^−1^ and outstanding stability, surpassing most of the previously reported catalysts, including the industrial benchmark SPERA catalyst developed by Chiyoda Corporation.^[^ ^26^ ^]^

## 2 Results and Discussion

### 2.1 Synthesis and Characterization of PtFeSn/AC

**Figure 1:**
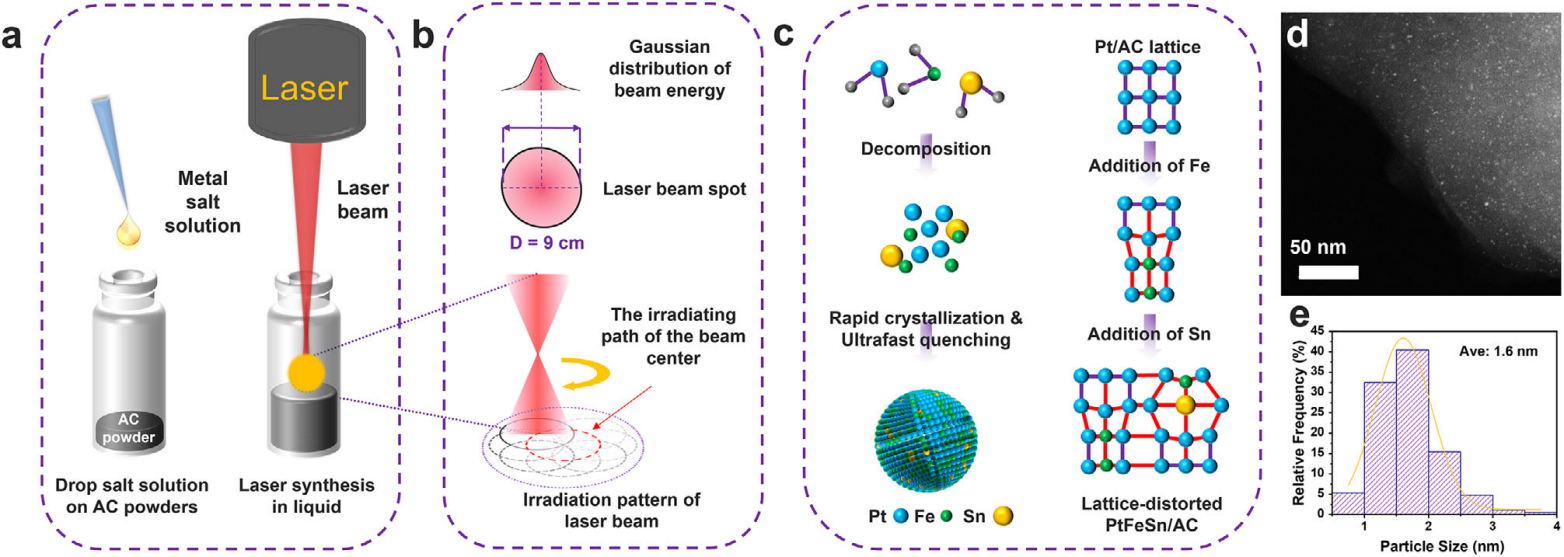
Schematic illustrations of a) the LSL method, b) irradiation pattern of laser beam, c) mechanism of alloy/AC formation and the generation of lattice distortion by LSL (left) and varying levels of lattice distortion due to the incorporation of Fe and Sn into the Pt nanoparticles (right). d) STEM images of PtFeSn/AC and e) the corresponding histogram of the particle size distribution.

The schematic illustration in **Figure** **1**a depicts the LSL method for synthesizing PtFeSn catalysts supported on AC. This process begins by mixing the metal precursor solution in an appropriate ratio of different metal cations with the AC support. After 15 min of sonication, the uniform mixture is subjected to laser irradiation for 42 s according to the programmed pattern shown in Figure 1b. The laser synthesis method achieves ultrahigh temperatures within nanoseconds through the photothermal effect causing metal precursor decomposition and reduction to metallic particles.^[^ ^27^, ^28^ ^]^ In our LSL process, due to solvent evaporation, rapid cooling occurs simultaneously in the liquid environment. As a result, due to the atomic radius difference among Pt (209 pm), Fe (194 pm), and Sn (217 pm), a distorted lattice is preserved by fast quenching, as illustrated in Figure 1c. The average size of the PtFeSn nanoparticles, with actual metal loading of 0.52 wt% Pt, 0.14 wt% Fe, and 0.04 wt% Sn (Table S1, Supporting Information), is ≈1.6 nm, as shown in Figure 1d,e. The small particle size and narrow size distribution are attributed to the rapid quenching in the liquid environment. In contrast, WI‐PtFeSn/AC, which was synthesized by the conventional wet impregnation and chemical reduction method, exhibits an average particle size of 2.2 nm and a broader particle size distribution (Figure S1, Supporting Information). Figure S2 (Supporting Information) shows the element mapping of the as‐synthesized PtFeSn/AC, confirming the homogeneous distribution of Pt, Fe and Sn.

**Figure 2:**
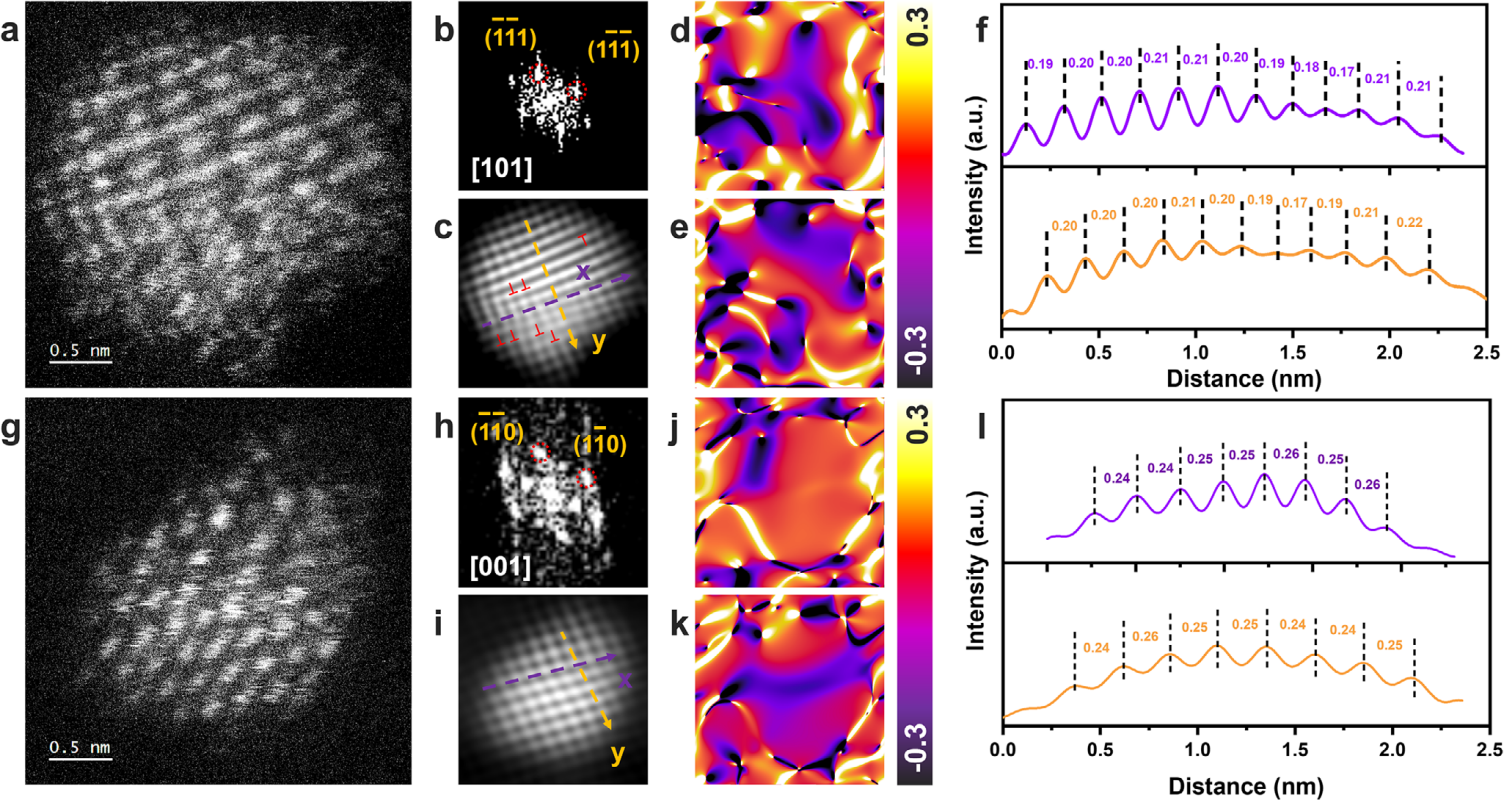
Microscopy and strain characterization of PtFeSn/AC and PtFe/AC nanoparticles. a,g) STEM images of an individual PtFeSn and PtFe nanoparticle, and their corresponding b,h) FFT patterns, c,i) inverse FFT patterns, d,e,j,k) strain distribution in geometric phase images of Ɛ_xx_ (d,j) and Ɛ_yy_ (e,k) direction, and f,l) HAADF intensity profiles and measurement of atomic spacing labeled in (c,i).

The HAADF‐STEM images of PtFeSn/AC and PtFe/AC are shown in **Figure** **2**a,g. The corresponding fast Fourier transform (FFT) patterns oriented close to the [1 0 1] and [0 0 1] zone axis, are displayed in Figure 2b,h, together with their inverse FFT (IFFT) patterns (Figure 2c, 2i) processed based on the circled diffraction spots in Figure 2b,h. Lattice sites marked with “T” denote dislocations. Geometrical phase analysis (GPA),^[^ ^29^, ^30^ ^]^ based on atomic‐resolution HAADF‐STEM data from the (−1 1 1), (1 −1 −1) and (−1 −1 0), (1 −1 0) lattice planes, provides quantitative strain maps for *Ɛ_xx_* (Figure 2d,j) and *Ɛ_yy_* (Figure 2e,k). Warm colors on the maps indicate expansive lattice deformation, while cool colors represent lattice contraction relative to the reference. The varying color patterns in the GPA maps confirm the presence of strain within the PtFeSn/AC nanoparticles. Figure 2f presents the intensity profiles along line x (purple) and line y (orange), both indicating varied d‐spacing for PtFeSn (−1 −1 1) and (1 −1 −1) lattice planes. On the contrary, the HAADF‐STEM image of PtFe/AC shows more ordered atomic arrangement than that of PtFeSn/AC. The IFFT pattern reveals a regular crystal plane with fewer lattice distortions and defects. The GPA of PtFe/AC further demonstrates a more uniform strain distribution compared to PtFeSn/AC, with no significant dilatation or constriction, especially in the *Ɛ_xx_* direction, implying a weaker strain effect in PtFe/AC. The intensity profiles also show less variation in lattice distance. The same analyses were conducted for other samples including WI‐PtFeSn/AC, PtSn/AC and Pt/AC. As shown in Figures S3–S5 (Supporting Information) no obvious lattice distortion can be observed and the strain mapping reveals only a mild strain distribution in both *Ɛ_xx_* and *Ɛ_yy_* directions. In contrast, the laser‐synthesized PtFeSn/AC exhibits greater distortion, attributed to the rapid‐quenching effect of LSL and the higher degree of atomic misfit. The elemental distribution results (Figures S6 and S7, Supporting Information) indicate that Pt and Fe are co‐located in the same nanoparticles in PtFeSn/AC, PtFe/AC and WI‐PtFeSn/AC, while Sn is more homogeneously spread. As the loading of Sn is very low at 0.04–0.05 wt%, it is challenging to image its exact location precisely. Nonetheless, its presence in the metal nanoparticles is evidenced by the enhanced strain comparing to the GPA mappings of samples with and without Sn (Figure 2; Figures S4 and S5, Supporting Information).

**Figure 3:**
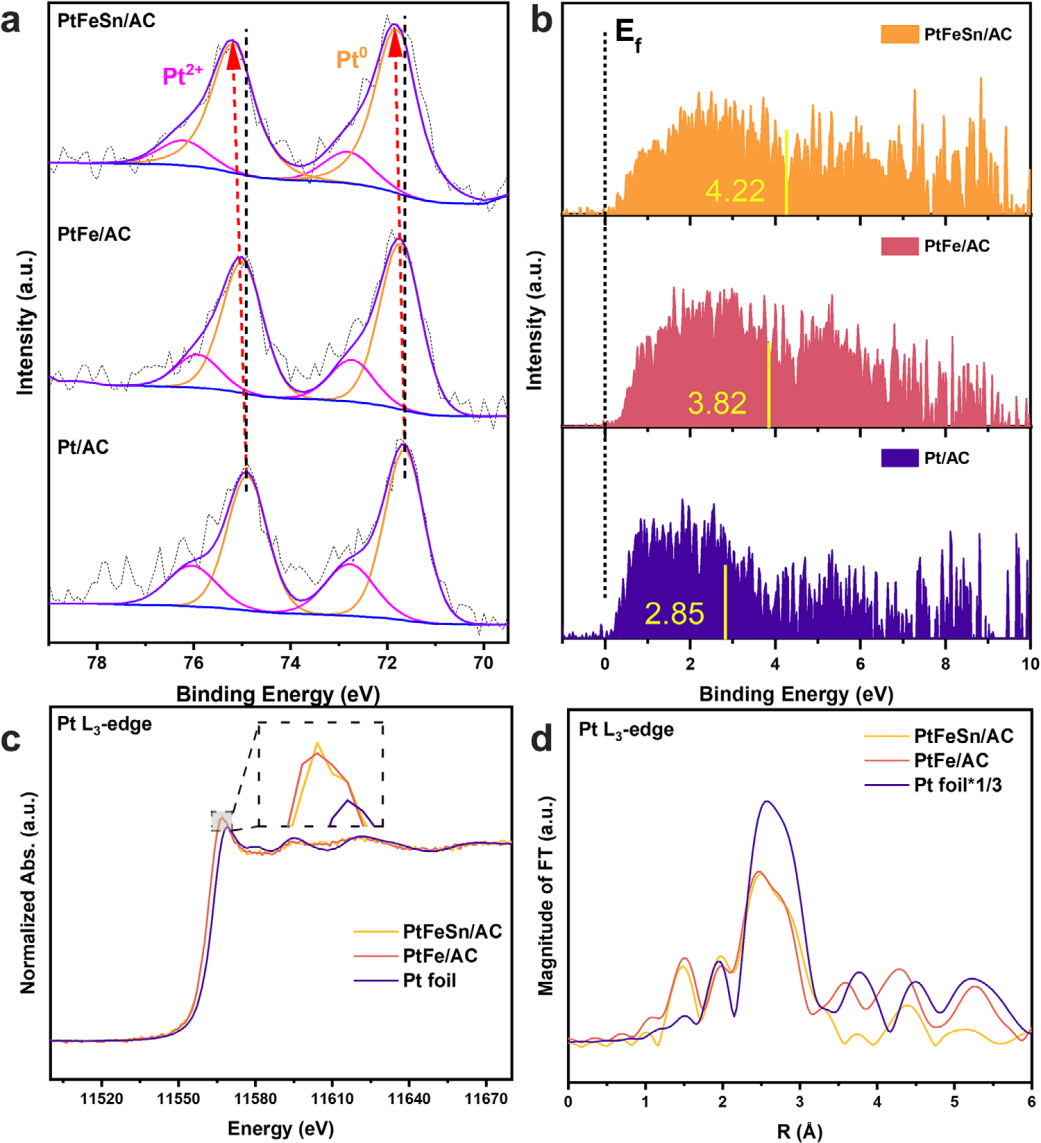
a) Pt 4f XPS spectra of PtFeSn/AC, PtFe/AC, Pt/AC. b) Normalized valence band spectra (VBS) of PtFeSn/AC, PtFe/AC, Pt/AC measured by X‐ray photoelectron spectroscopy (XPS). c) Pt L_3_‐edge XANES spectra and d) Fourier‐transforms of k^3^‐weight Pt L_3_‐edge for PtFeSn/AC, PtFe/AC catalysts and Pt foil.

X‐ray photoelectron spectroscopy (XPS) of freshly reduced samples was used to investigate the electronic structure of PtFeSn/AC. As shown in **Figure** **3**a, compared to Pt/AC, the observed positive shift to higher binding energy of the Pt 4f peak for PtFeSn/AC and PtFe/AC suggests a decrease in the electron density of Pt. As reported, the shift of Pt 4f to higher binding energy also indicates the introduction of compressive strain, with a more pronounced positive shift reflecting a higher level of compressive strain.^[^ ^29^, ^31^, ^32^ ^]^ The compressive strain in PtFe/AC originates from the disordered distribution of Fe atoms, which leads to random coordination between Pt and Pt, as well as Pt‐Fe interactions. The XPS peak shift confirms that compressive strain predominates in PtFeSn/AC. The Pt‐Pt/Fe atoms are mostly compressed due to the introduction of larger Sn atom which is consistent with the GPA map results shown in Figure 2d,f. The presence of Pt^2+^ in the samples is due to partial oxidation during storage. As a result, this should lead to an increased overlap of the *d* orbital and subsequent broadening of the *d* band. The *d*‐band center shifts to a lower energy to maintain the same filling degree, consequently, the density of states near the Fermi level decreases, making it more energetically difficult to remove core electrons, thereby increasing the metallic Pt binding energy.^[^ ^31^, ^33^, ^34^ ^]^ As shown in Figure 3b, the *d*‐band center (marked by the vertical yellow lines) is the weighted center of the valence band spectra (VBS), derived after the subtraction of the Shirley‐type baseline, integration, and normalization.^[^ ^35^, ^36^ ^]^ Notably, a significant downshift of the *d*‐band center is observed for PtFeSn/AC (4.22 eV) compared to Pt/AC (2.85 eV), confirming the lattice compression. It was reported that this downshift leads to weaker adsorption of O‐containing intermediates during oxygen reduction reaction,^[^ ^37^ ^]^ attributed to the increased filling of absorbate‐metal antibonding states according to the *d*‐band theory.^[^ ^38^ ^]^ It is expected that this phenomenon will also cause a weaker adsorption energy of some intermediates during MCH dehydrogenation.

The X‐ray absorption near edge structure (XANES) of Pt L_3_‐edge, shown in Figure 3c, reveals that the white line peak of both PtFeSn/AC and PtFe/AC exhibits a higher intensity compared to Pt foil, indicating an elevated valence state, aligning with XPS results. The Fourier‐transformed extended X‐ray absorption fine structure (FT‐EXAFS) spectra of Pt L_3_‐edge (Figure 3d) reveal that the average Pt‐M bond length in PtFeSn/AC becomes shorter, following the order of Pt foil > PtFe/AC ≈ PtFeSn/AC. This trend is further identified via the corresponding wavelet transform (WT) plots (Figure S8, Supporting Information). The slight contraction of the Pt‐Pt/Fe bond is derived from the local compressive strain. Meanwhile, the peak height is positively correlated to the coordination number.^[^ ^39^ ^]^ Thus, the lower peak intensity of PtFeSn/AC compared to PtFe/AC suggests a more unsaturated coordination environment. The main correlative shell fitting results are summarized in Table S2 (Supporting Information), confirming that the Pt‐Pt and Pt‐M bond lengths were 2.73 and 2.66 Å, respectively, which are smaller than those of PtFe/AC (2.74 Å for Pt‐Pt bond and 2.70 Å for Pt‐Fe bond). The coordination number of Pt‐Pt and Pt‐M was 4.6 and 2.2 for PtFeSn/AC compared to 6.7 and 3.0 for PtFe/AC. The corresponding curve fitting of EXAFS results are presented in Figure S9 (Supporting Information). In conclusion, a combination of HAADF‐STEM, XPS, and XAFS analyses revealed that the lattice distortion in PtFeSn/AC induces lattice compression. The compressive strain results in shorter bond lengths, higher Pt valence states, and an unsaturated coordination environment, thereby resulting in the downshift of the *d*‐band center, as explained by the *d*‐band center theory.^[^ ^40^ ^]^

### 2.2 Catalytic Performance for MCH Dehydrogenation

**Figure 4:**
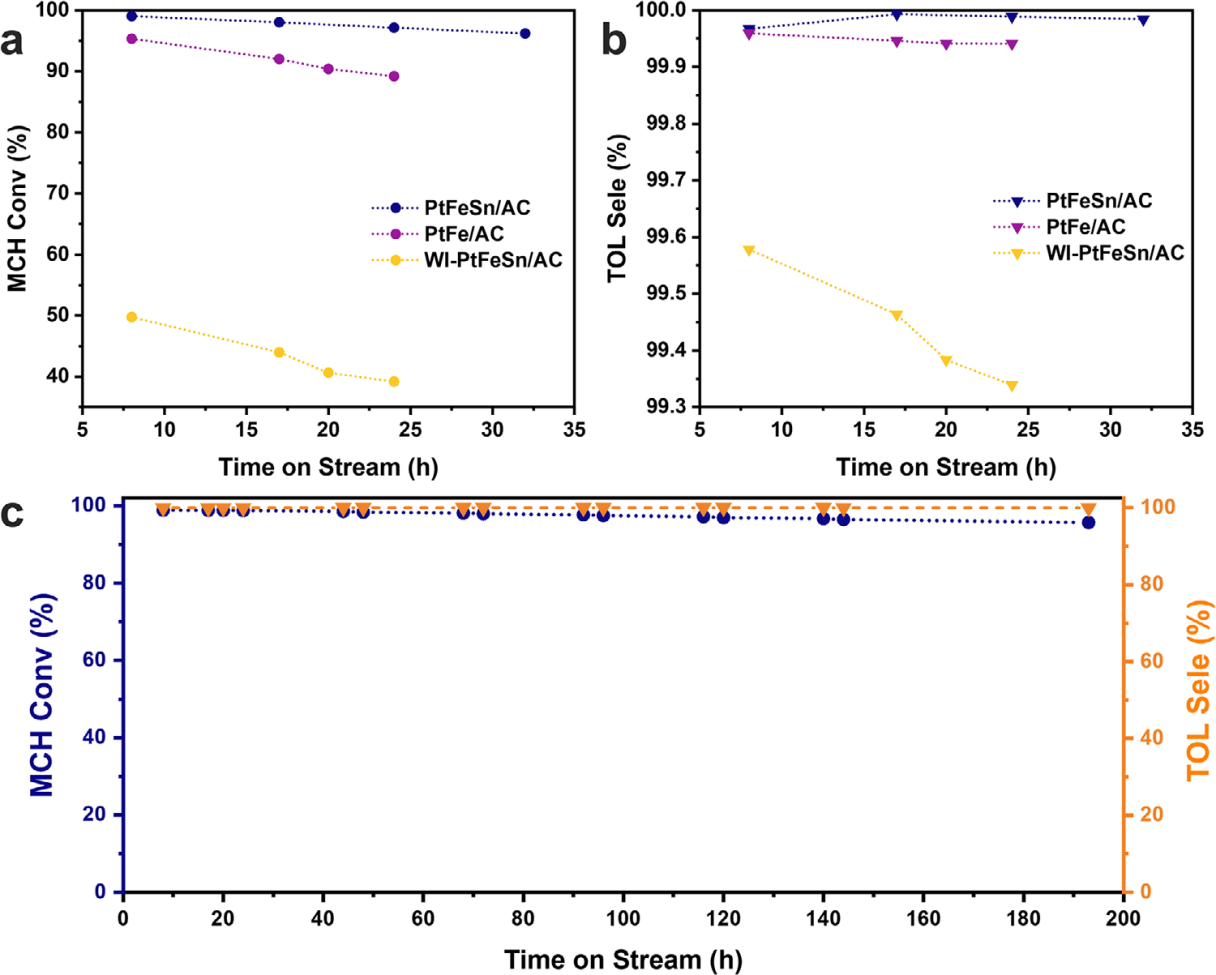
Evaluation of MCH dehydrogenation performance of the as‐prepared catalysts. a,b) MCH conversion and TOL selectivity curves of PtFeSn/AC, PtFe/AC, and WI‐PtFeSn/AC (MCH flow rate at 25 °C, 1.5 atm = 0.24 mL min^−1^, weight of catalyst = 400 mg WHSV = 27.7 h^−1^, T = 375 °C). c) MCH conversion and TOL selectivity curves of PtFeSn/AC for long‐term stability test (MCH flow rate at 25 °C, 1.5 atm = 0.12 mL min^−1^, weight of catalyst = 400 mg WHSV = 13.9 h^−1^, T = 375 °C).

To balance efficient catalyst evaluation with experimental feasibility, two different WHSVs were used for activity and stability tests. A higher WHSV of 27.7 h⁻¹ was applied during activity screening to accelerate reaction rates and highlight performance differences among catalysts within a short duration. These conditions promote faster coke accumulation, enabling rapid identification of active candidates. For long‐term stability evaluation, a lower WHSV of 13.9 h⁻¹ was selected. Although higher than typical industrial values (≈5 h⁻¹ for MCH dehydrogenation), this condition represents a practical compromise that enables accelerated aging while maintaining relevance to continuous operation. Similar dual‐WHSV strategies have been adopted in previous catalyst development studies.^[^ ^8^, ^13^, ^41^ ^]^ The effect of lattice strain on MCH dehydrogenation performance is clearly observed over PtFeSn/AC, PtFe/AC, and WI‐PtFeSn/AC as shown in **Figure** **4**a,b. In the absence of Fe or both Fe and Sn (Figure S12, Supporting Information), PtSn/AC (85.1%, 0.37% h^−1^) and Pt/AC (94.8%, 0.46% h^−1^) exhibit lower initial MCH conversion and a faster deactivation rate compared to PtFe/AC (95.3%, 0.38% h^−1^) and PtFeSn/AC (99.0%, 0.11% h^−1^), which is attributed to the absence of lattice distortion. The WI‐PtFeSn/AC prepared by wet impregnation followed by chemical reduction exhibits the poor performance of low initial MCH conversion (49.8%) and fast catalyst deactivation rate (0.67% h^−1^). This is probably due to the larger catalyst particle sizes and the absence of lattice strain. To investigate the optimal ratio of Pt‐Fe‐Sn, several catalysts with varying elemental compositions in the precursor solutions were prepared, as shown in Figure S11 (Supporting Information). The catalyst with a Pt:Fe:Sn atomic ratio of 1:0.75:0.125 exhibits the best dehydrogenation performance during the screening test. The particle aggregation was observed in the spent WI‐PtFeSn/AC (Figure S13, Supporting Information), with a 60% increase in average particle size from 2.2 to 3.6 nm. On the contrary, the size difference between fresh and spent LSL catalysts is much smaller (Figure S14, Supporting Information). The average metal particle size of spent PtFeSn/AC remains as small as 1.4 nm after 24 h of reaction. This indicates a stronger interaction between the metal particles and AC support in LSL catalysts.

For practical applications, long‐term catalyst stability and recyclability are important factors. A stability study was carried out at a WHSV of 13.9 h^−1^. Figure 4c shows that the PtFeSn/AC catalysts still maintain over 95% MCH conversion and an ultrahigh TOL selectivity (99.96%) after 193 h at 375 °C. The slight decrease in activity might be due to coke formation, which can partially block active site.^[^ ^42^ ^]^ The hydrogen evolution rate of PtFeSn/AC reaches 2625 mmol g_Pt_^−1^ min^−1^ under harsh reaction condition (WHSV = 27.7 h^−1^). In addition, the STEM imaging and strain mapping of used PtFeSn/AC confirm the preservation of lattice distortion after 24 h MCH dehydrogenation under harsh reaction conditions (Figure S15, Supporting Information). Table S3 (Supporting Information) compares the activity and stability of our PtFeSn/AC catalyst with those of Pt‐based catalysts reported in the literature. It shows that PtFeSn/AC is one of the most promising catalysts reported so far.

**Figure 5:**
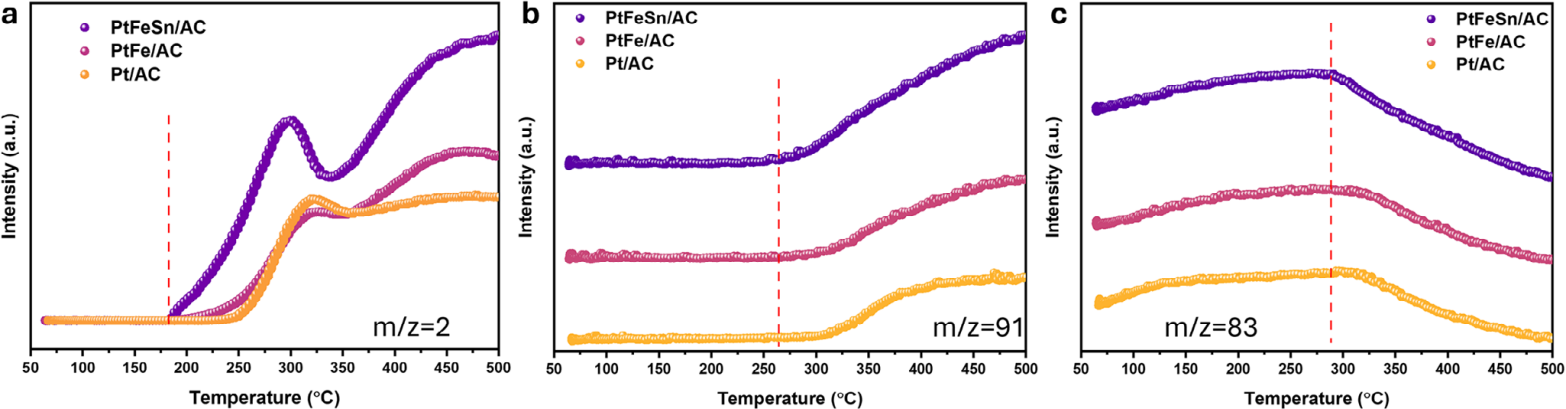
MCH‐TPSR analysis, a) H_2_ curves, b) TOL curves, and c) MCH curves.

Additionally, temperature‐programmed surface reaction (MCH‐TPSR) was conducted for LSL‐prepared catalysts and the results align with the dehydrogenation performance of catalysts. As shown in **Figure** **5**, the H_2_ evolution onset temperature of PtFeSn/AC and PtFe/AC are both ≈180.0 °C, significantly lower than that of Pt/AC (240.0 °C), indicating that alloying Pt with Fe reduces C‐H bond activation energy.^[^ ^43^ ^]^ PtFeSn/AC exhibits the lowest TOL desorption onset temperature of 275.0 °C, compared to PtFe/AC (287.0 °C) and Pt/AC (296.1 °C), suggesting that Sn facilitates TOL desorption and supress the coke formation.^[^ ^12^, ^44^ ^]^ The trend aligns with the MCH consumption temperature of these catalysts. The slight increase in MCH concentration prior to reaching the reaction temperature could be attributed to MCH desorption with increasing temperature. In previous studies, TOL desorption was identified as the rate‐limiting step in MCH dehydrogenation, with strongly adsorbed TOL converting to coke and causing the catalyst degradation.^[^ ^14^, ^45^, ^46^, ^47^, ^48^, ^49^ ^]^ Our findings here suggest that the alloyed PtFeSn nanoparticles with lattice distortion and downshifted *d*‐band center leads to a weaker TOL adsorption energy and facile TOL desorption. The amounts of methane and benzene detected is insignificant for all LSL‐prepared catalysts during TPSR measurement. To analyze the relationship between the percentage of light coke and the level of lattice distortion, the thermalgravimetric analysis (TGA) was performed in air for the LSL‐prepared samples. As indicated in Figure S16 (Supporting Information), two stages of weight loss are observed in the TGA curves. Between 400 and 600 °C, the weight loss corresponds to the oxidation of activated carbon support. In the temperature range of 200 to 300 °C, a noticeable weight loss is observed in the used catalysts compared with fresh catalysts, confirming the formation of light coke over the catalyst surface.^[^ ^50^, ^51^ ^]^ PtFeSn/AC and PtFe/AC demonstrate stronger anti‐coking ability, with light coke weight loss of 1.65% and 2.12%, respectively, indicating the facile TOL desorption and side reaction suppression, aligning with the VBS and MCH‐TPSR results.

The temperature programmed oxidation (TPO) result of PtFeSn/AC (Figure S17, Supporting Information) shows a small peak located ≈250 °C, attributed to the oxidation of light coke, which appears only in the spent sample. Based on the TGA and TPO results, light coke removal and catalyst regeneration were performed under a mild condition. PtFeSn/AC was first tested at hasher condition of WHSV at 27.7 h^−1^ for 72 h to induce coke formation and then in situ regenerated in flowing air at 300 °C for 1 h, followed by in situ H_2_ reduction at 400 °C for 2 h. As shown in Figure S18 (Supporting Information), the regenerated PtFeSn/AC retains ≈98.6% of the initial MCH conversion of the fresh catalyst under same reaction condition. Meanwhile, TOL selectivity is above 99.95% both before and after regeneration. This result indicates that the catalyst was effectively regenerated through the combustion of light coke.

## 3 Conclusion

In summary, the PtFeSn/AC with a distorted lattice was successfully synthesized due to the atomic radii difference, and the fast quenching inherent to the LSL process. The PtFeSn/AC catalyst demonstrated stable MCH conversion and high TOL selectivity. HAADF‐STEM, GPA analysis, XPS, and EXAFS results revealed that lattice distortion is well distributed within the PtFeSn/AC. This distorted lattice induces an unsaturated coordination environment and lattice contraction, leading to more exposed active sites and a weaker interaction between the catalyst and coke precursors. Consequently, PtFeSn/AC reaches a hydrogen evolution rate of 2625 mmol g_Pt_^−1^ min^−1^ at WHSV of 27.7 h^−1^, outperforming most reported Pt‐based catalysts. Furthermore, the catalyst demonstrated exceptional durability, maintaining MCH conversion above 95% over 193 h time‐on‐stream, while delivering stable and ultrahigh TOL selectivity of over 99.96%. This findings from this study could guide the future design of alloy catalysts to achieve high performances.

## 4 Experimental Section

### Chemicals

The activated carbon (AC, H1‐G) powder was purchased from Foshan Porous Carbon Tech Co., Ltd. H_2_PtCl_6_ 6H_2_O, FeCl_3_ 6H_2_O, SnCl_2_ 2H_2_O, hexane (≥95%), ethylene glycol, ethanol (≥95%) and methylcyclohexane (MCH, anhydrous, ≥99%) were purchased from Sigma‐Aldrich.

### PtFeSn/AC Catalysts Prepared by Laser Synthesis in Liquid (LSL) Method

100 mg of activated carbon support was put in a 5 mL glass vial. H_2_PtCl_6_ 6H_2_O, FeCl_3_ 6H_2_O, SnCl_2_ 2H_2_O were dissolved individually in ethanol to prepare solutions with a concentration of 10 mM. Subsequently, the metal precursor solutions were dropped into the glass vial with an atomic ratio of 1: 0.75: 0.125 (Pt:Fe:Sn), and the resultant mixture was sonicated for 15 min to ensure uniform dispersion. All sample solutions were irradiated by a laser beam with consistent parameters for two times (1^st^ time: 28 s, and 2^nd^ time: 14 s) under an ambient environment and with 2 mL of hexane added to seal top of the solution before each time irradiation. The parameters of the laser beam were set to 5 mm^−1^ scan rate, 55 W output power, 2700 kHz frequency, 2 ns Q pulse and 9 mm beam spot, and 29 cm beam gun height (from beam gun to the sample stage), in alignment with the commercial laser source (JPT‐M7‐100 W). After the LSL process, the alloy nanoparticles were successfully synthesized on the surface of the AC support. Subsequently, the catalysts were washed several times with ethanol followed by and drying at 60 °C in the oven overnight.

### WI‐PtFeSn/AC Catalysts Prepared by Wet Impregnation and Chemical Reduction Method

Briefly, 1.0 g of AC support was dispersed in 10 mL of ethanol and stirred for 20 min in a round bottom flask with condenser. Then the individual metal precursor solutions (10 mM) were added dropwise with an atomic ratio of 1: 0.75: 0.125 (Pt:Fe:Sn) and the mixture was stirred for 30 min. Then, 10 mL of ethylene glycol was added and the mixture was heated at 100 °C for 4 h under continuous stirring in the air. The solid sample was washed with ethanol several times following with drying at 60 °C in the oven overnight.^[^ ^52^ ^]^

### Characterization

The TEM images, STEM‐EDS mapping, and line scan were acquired in a JEOL 2100F. The STEM images were acquired in a JEOL ARM‐200F microscope equipped with a JEOL delta aberration corrector and a cold field emission gun operating at 200 kV. Geometric phase analysis (GPA) is a digital signal processing method for quantifying displacements and strain fields at atomic resolution by utilizing the real‐space and Fourier‐space information of HRTEM or HAADF‐STEM image. According to the geometric phase analysis method created by Hÿtch et al,^[^ ^30^ ^]^ two diffraction spots which can define the 2D HAADF‐STEM image in real space were chosen, and the lattice distortion of the high‐resolution phase can be measured by this reference. An FRWR tool plugin from the Humboldt University of Berlin was used, which can be plugged into the DigitalMicrograph (Gatan) software to establish the strain mapping of HAADF‐STEM images. An in‐plane strain (*Ɛ_xx_*) field was obtained to show the strain distribution. The X‐ray photoelectron spectroscopy (XPS) data were collected in an X‐ray photoelectron spectrometer (Kratos AXIS Supra), with non‐monochromatized Al K𝛼 X‐rays as the excitation source. The Pt L_3_‐edge X‐ray absorption spectra were collected in the XAFS beamline facility at the Singapore Synchrotron Light Source using the transmission mode. Acquired EXAFS data were processed according to standard procedures using the ATHENA module implemented in the IFEFFIT software package. The k3‐weighted Fourier transforms for the Pt L_3_‐edge EXAFS data were performed over a k‐range 3–12 Å^−1^ using a Hanning‐shaped window. The k and R ranges for the fitting of Pt L_3_‐edge of the EXAFS data were set as 3–12 Å^−1^ and 1.1–3.2 Å, respectively. The MCH‐TPSR spectra were acquired in a MicrotracBEL‐BELCAT II. Before the test, 100 mg of the catalysts were pretreated at 400 °C under a 5% H_2_/Ar atmosphere for 2 h. The sample was dried and pretreated by heating from room temperature to 300 °C at a rate of 10 °C min^−1^ under a helium flow of 50 mL min^−1^ for 1 h. The sample was then cooled to 50 °C, and MCH gas was introduced until adsorption saturation was achieved. Subsequently, the helium flow was maintained at 50 mL min^−1^ to purge the system for 1 h, removing any physically adsorbed MCH gas from the surface. Finally, the sample was heated to 500 °C at a rate of 10 °C min^−1^ in a 50 mL min^−1^ flow of MCH gas, and the evolved gases were analyzed using a mass spectrometry detector. The Temperature Programmed Oxidation (TPO) was conducted with 0.1 g of the catalyst in an Autochem II 2920 (Micrometrics, USA) instrument equipped with a TCD detector. The samples were heated to 800 °C at the rate of 5 °C min^−1^ under 50 mL min^−1^ airflow. The ICP‐OES results were acquired from the iCAP 7400 (Thermo, Waltham, USA) instrument. The TGA was conducted in a Mettler Toledo TGA/DSC1 instrument. The samples were heated to 900 °C at the rate of 10 °C min^−1^ under 50 mL min^−1^ airflow.

### Methylcyclohexane Dehydrogenation Reaction

The catalytic dehydrogenation of MCH was carried out in a fixed‐bed tubular stainless‐steel reactor with an internal diameter of 3/8 inch at 1.5 bar. In each reaction run, 0.4 g of catalyst was loaded into the constant temperature zone of the reactor and purged with 100 mL min^−1^ pure N_2_ flow for 30 min. The reactor temperature was increased to 400 °C under pure H_2_ flow (200 mL min^−1^) and maintained at this temperature for 2 h to ensure the complete reduction of the catalysts. After that, the reactor temperature was reduced and maintained at 375 °C. Liquid MCH was injected into the reactor by a KX204‐001 pump (Nihon Seimitsu Kagaku). Both the inlet and outlet tubing were heated at 200 and 160 °C, respectively, by a thermal tape to maintain the gas phase of MCH and TOL. The condensed liquid product of the reactor was analyzed by a gas chromatograph (Shimadzu) equipped with a flame ionization detector (FID). The conversion, selectivity, and hydrogen evolution rate (mmol g*_Pt_* ^−1^ min^−1^) were calculated as follows:

(1) $$Conversion_{MCH}\;\left(\%\right)=\frac{Moles\;of\;initial\;MCH-\;Moles\;of\;residual\;MCH}{Moles\;of\;initial\;MCH}\;\times 100$$

(2) $$Selectivity_{TOL}\;\left(\%\right)=\frac{Moles\;of\;product\;TOL}{Moles\;of\;initial\;MCH-\;Moles\;of\;residual\;MCH}\;\times 100$$

(3) $$\begin{array}{c} H_{2}\;evolution\;rate\;\left(mmol\;\;g_{Pt}^{-1}\;\;min^{-1}\right)=\\ \frac{Flow_{MCH}\;\left(mmol\;\;min^{-1}\right)\times Conversion_{MCH}\;\left(\%\right)\times Selectivity_{TOL}\left(\%\right)\times 3}{Pt\;weight\;\left(g\right)} \end{array}$$

## Conflict of Interest

The authors declare no conflict of interest.

## Supporting information



Supporting Information

## Data Availability

The data that support the findings of this study are available from the corresponding author upon reasonable request.
