# Supplementary material for: Laser Synthesis in Liquid Induced Lattice Distortion in PtFeSn/Activated Carbon for Enhanced Methylcyclohexane Dehydrogenation
Source: Small Methods. 2025 Jun 23;10(2):2500474. doi: 10.1002/smtd.202500474 (PMC12825338; doi:10.1002/smtd.202500474)
Supplement: Supplementary file 1 — Supporting Information [file SMTD-10-2500474-s001.docx]

Laser Synthesis in Liquid Induced Lattice Distortion in PtFeSn/Activated Carbon for Enhanced Methylcyclohexane Dehydrogenation

Zheng Wang,[a] Hossein Akhoundzadeh,[a] Mudi Wu,[a] Mingwu Tan,[c] Yizhong Huang,[b]* Rong Xu,[a]*

[a] School of Chemistry, Chemical Engineering and Biotechnology, Nanyang Technological University, 62 Nanyang Drive, 637459 Singapore

[b] School of Materials Science and Engineering, Nanyang Technological University, 50 Nanyang Avenue, Singapore, 639798 Singapore

[c] Institute of Sustainability for Chemicals, Energy and Environment (ISCE2), Agency for Science, Technology and Research (A*STAR), 1 Pesek Road, Jurong

*Corresponding authors.

E-mail: yzhuang@ntu.edu.sg (Yizhong Huang), rxu@ntu.edu.sg (Rong Xu)


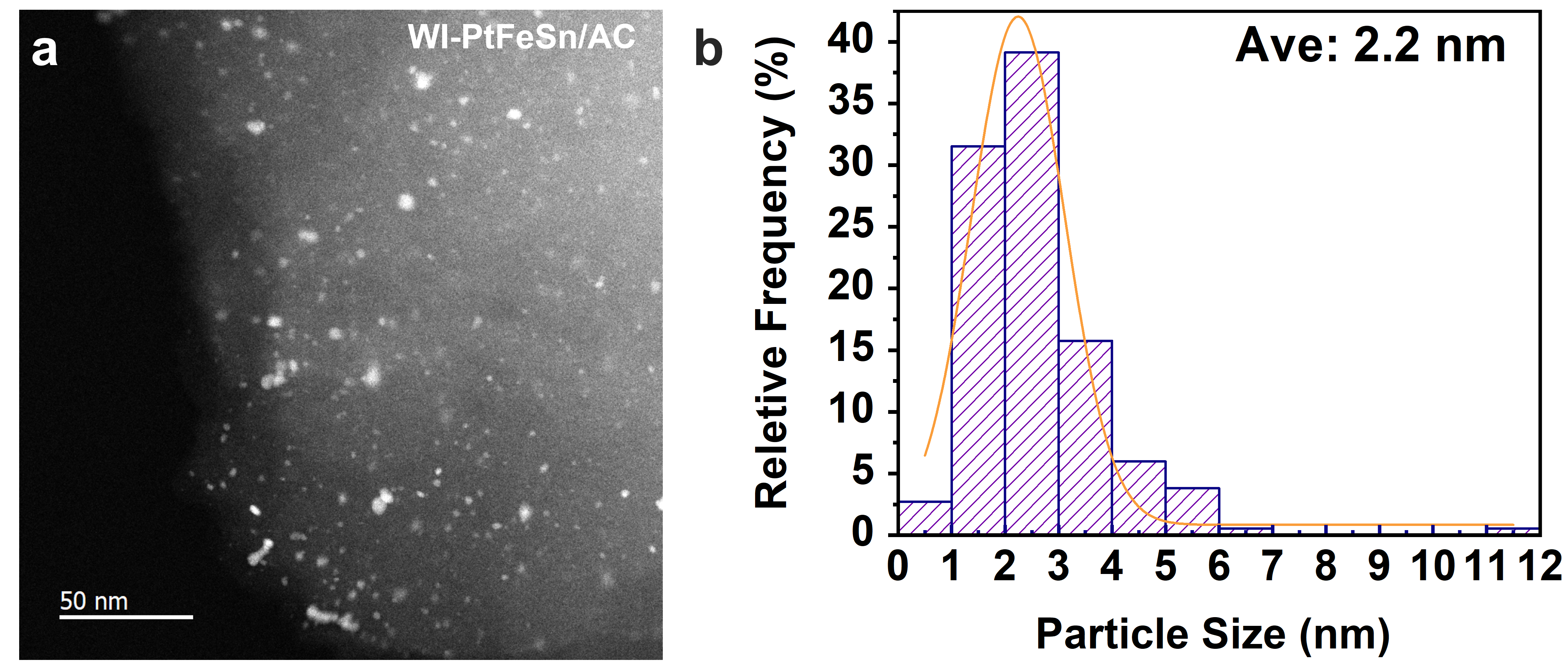


**Figure S1.** a) HAADF-STEM images of WI-PtFeSn/AC nanoparticles. b) The corresponding histograms of the statistics of the nanoparticle size distribution in a).


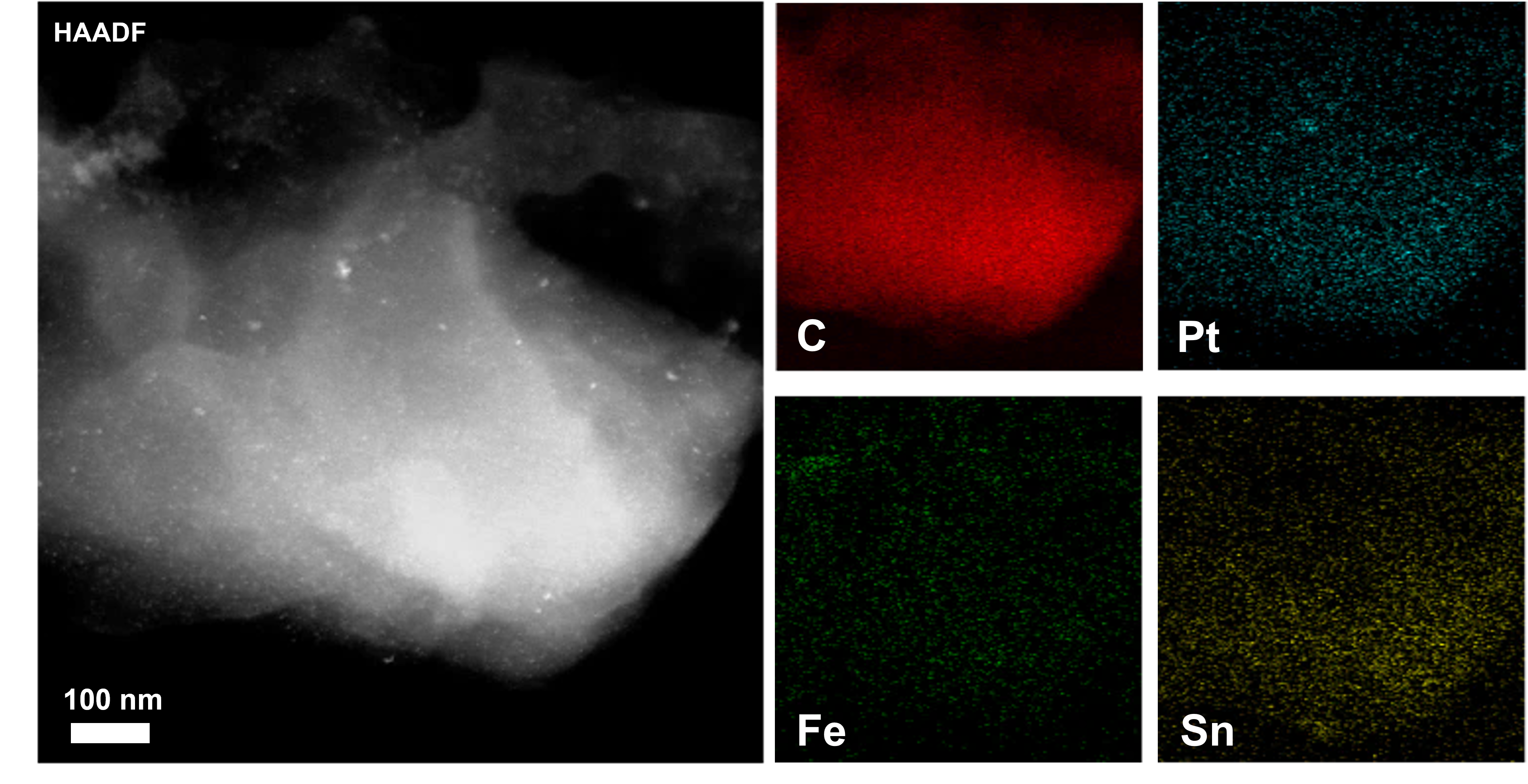


**Figure S2.** STEM-EDS elemental mapping of PtFeSn/AC.


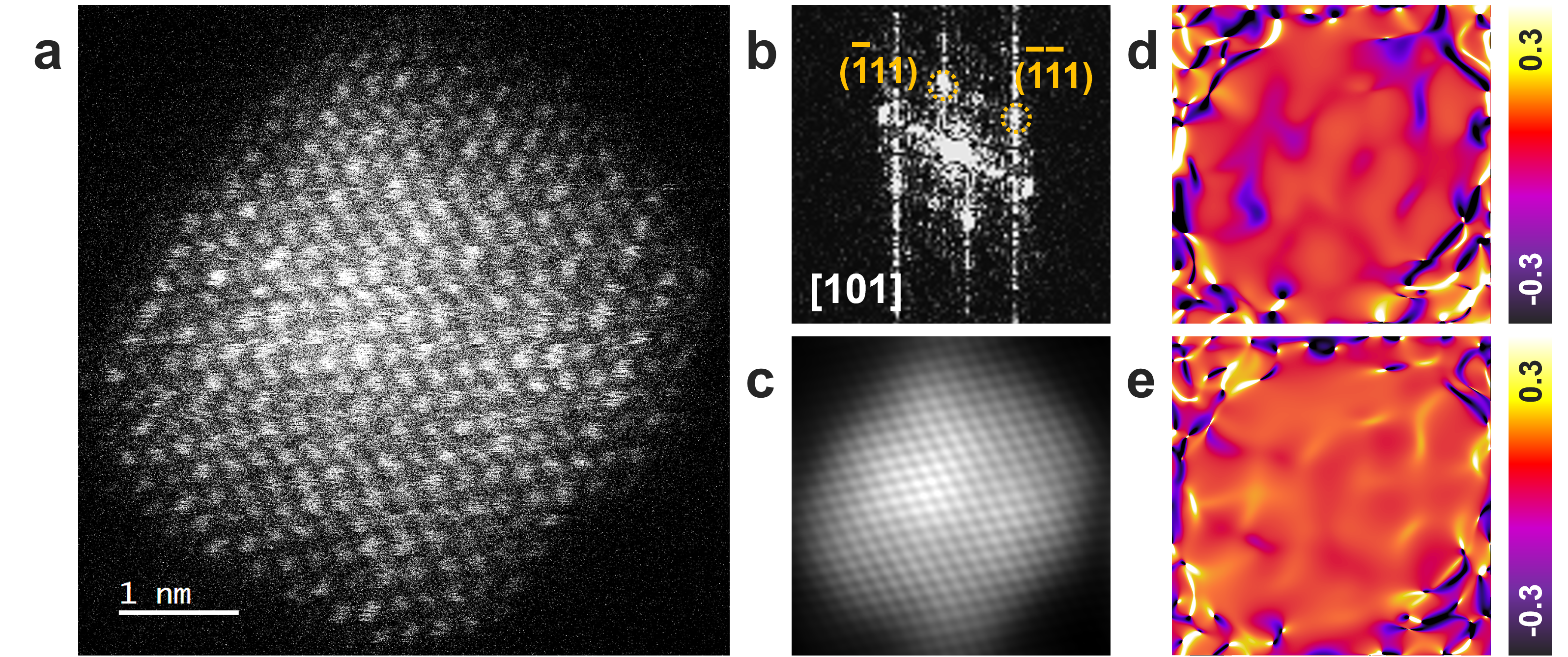


**Figure S3.** Microscopy and strain characterization of WI-PtFeSn/AC nanoparticle. a) STEM images of WI-PtFeSn/AC nanoparticle. b) Corresponding Fast Fourier transfer (FFT) pattern of the nanoparticle. c) Inverse FFT (IFFT) patterns of b). d, e) The strain distributions in geometric phase image of Ɛ_xx_ and Ɛ_xy_ direction of the nanoparticle.


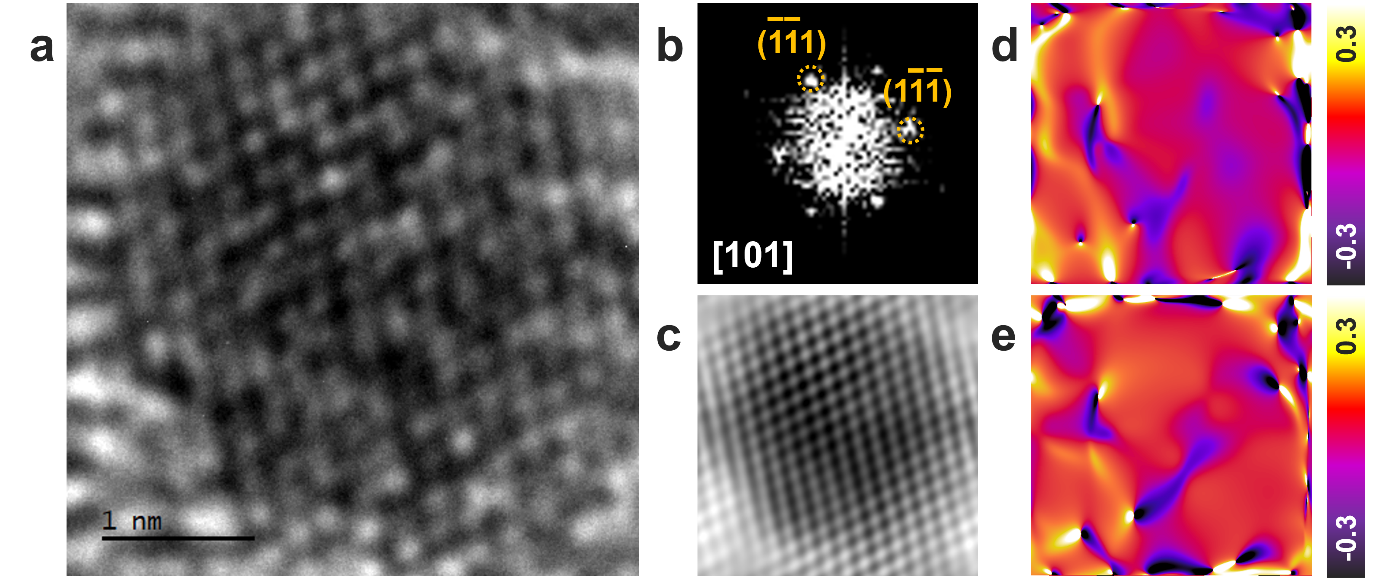


**Figure S4.** Microscopy and strain characterization of PtSn/AC nanoparticle. a) HRTEM images of PtSn/AC nanoparticle. b) Corresponding Fast Fourier transfer (FFT) pattern of the nanoparticle. c) Inverse FFT (IFFT) patterns of b). d, e) The strain distributions in geometric phase image of Ɛ_xx_ and Ɛ_yy_ direction of the nanoparticle.


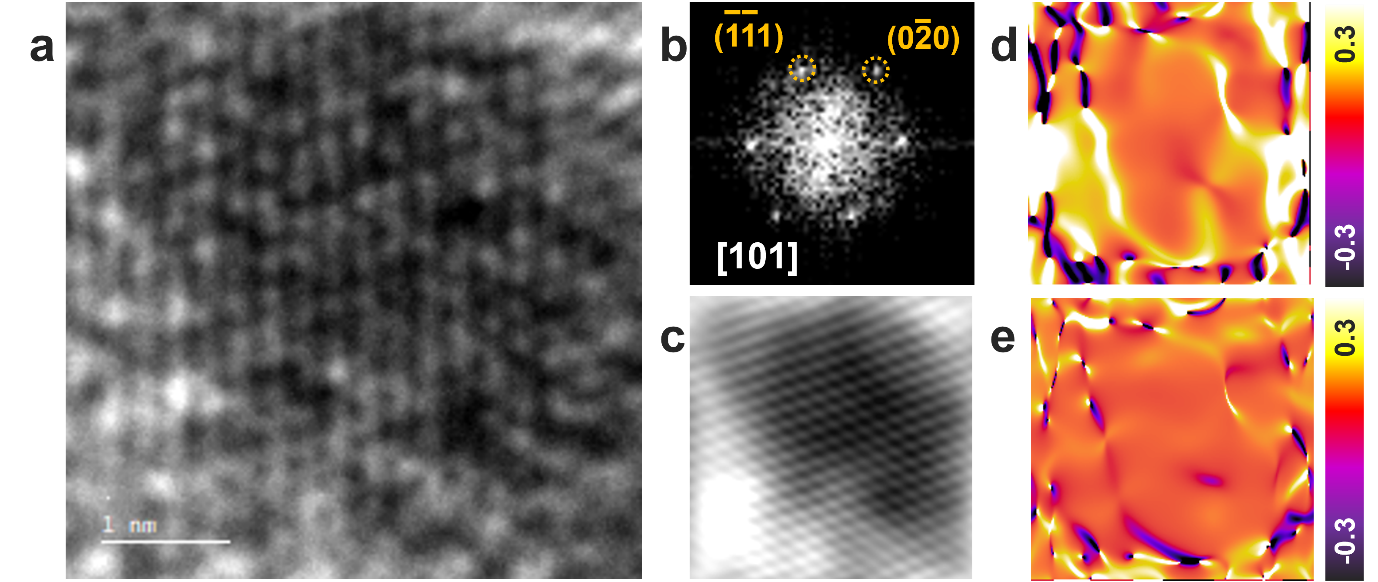


**Figure S5.** Microscopy and strain characterization of Pt/AC nanoparticle. a) HRTEM images of Pt/AC nanoparticle. b) Corresponding Fast Fourier transfer (FFT) pattern of the nanoparticle. c) Inverse FFT (IFFT) patterns of b). d, e) The strain distributions in geometric phase image of Ɛ_xx_ and Ɛ_yy_ direction of the nanoparticle.


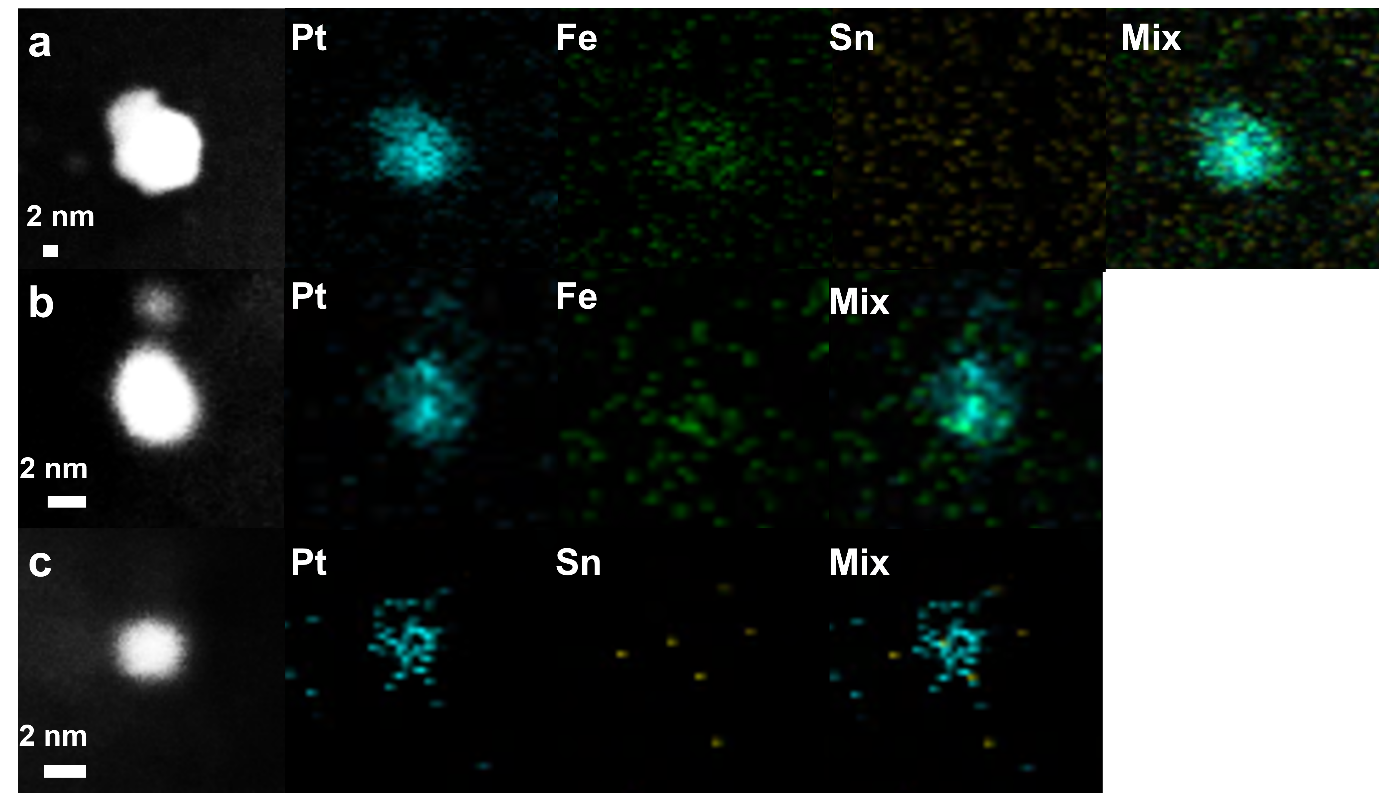


**Figure S6**. a-c) STEM-EDS mapping of PtFeSn/AC, PtFe/AC, PtSn/AC nanoparticles.


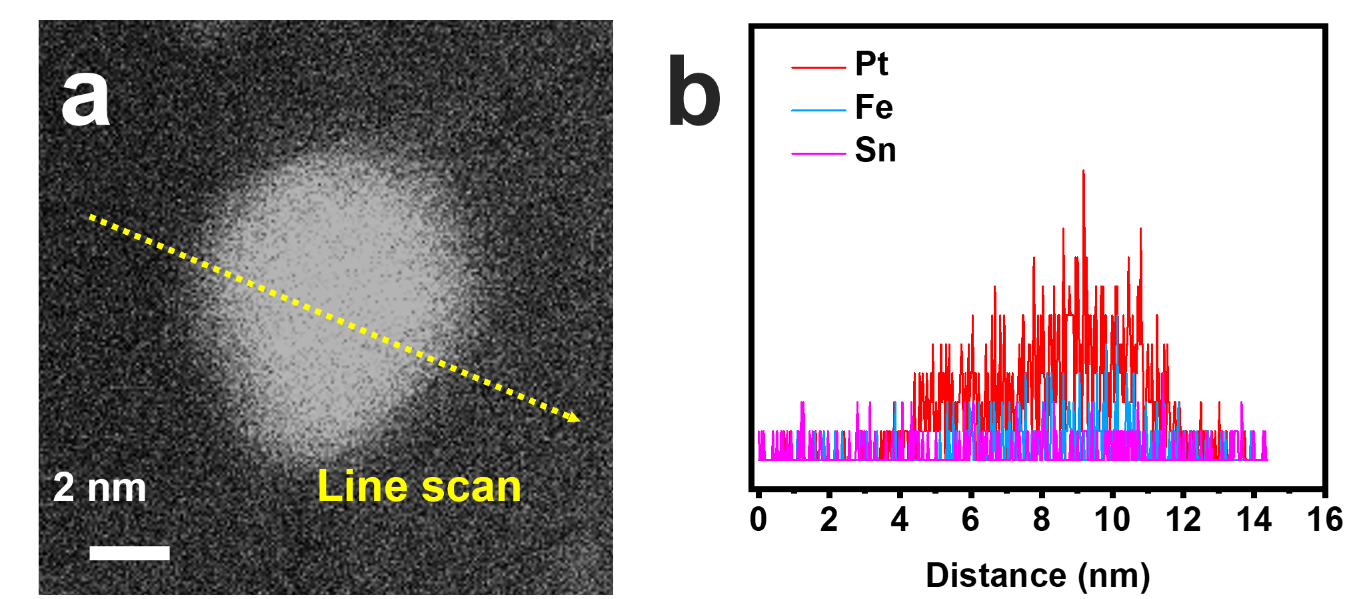


**Figure S7.** Microscopy characterization of wet impregnation synthesized NPs. a) High-resolution STEM image of WI-PtFeSn/AC nanoparticles. b) The corresponding STEM-EDS line-scan profiles.


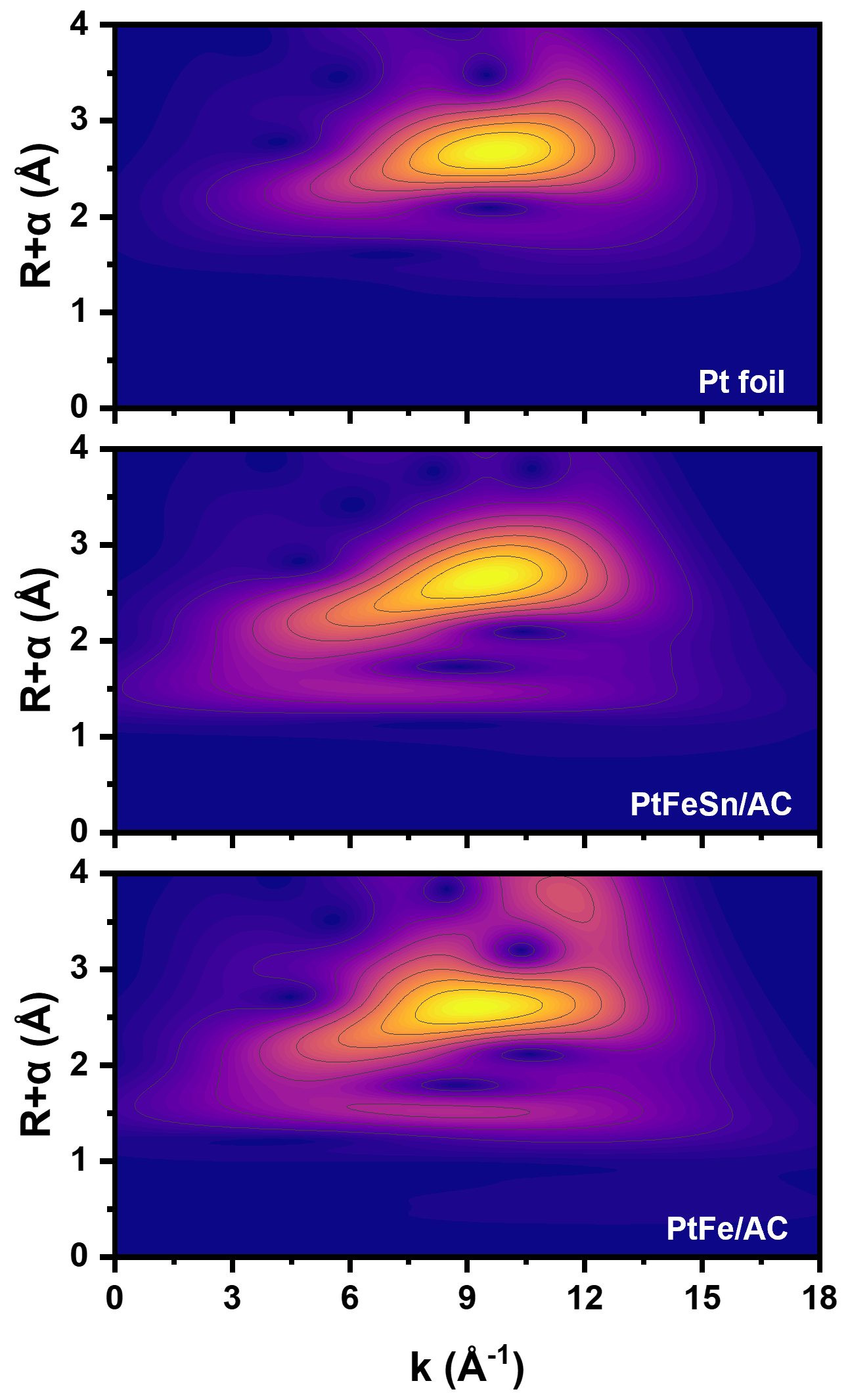


**Figure S8.** Wavelet transform (WT) plots of Pt Foil, PtFeSn/AC and PtFe/AC.


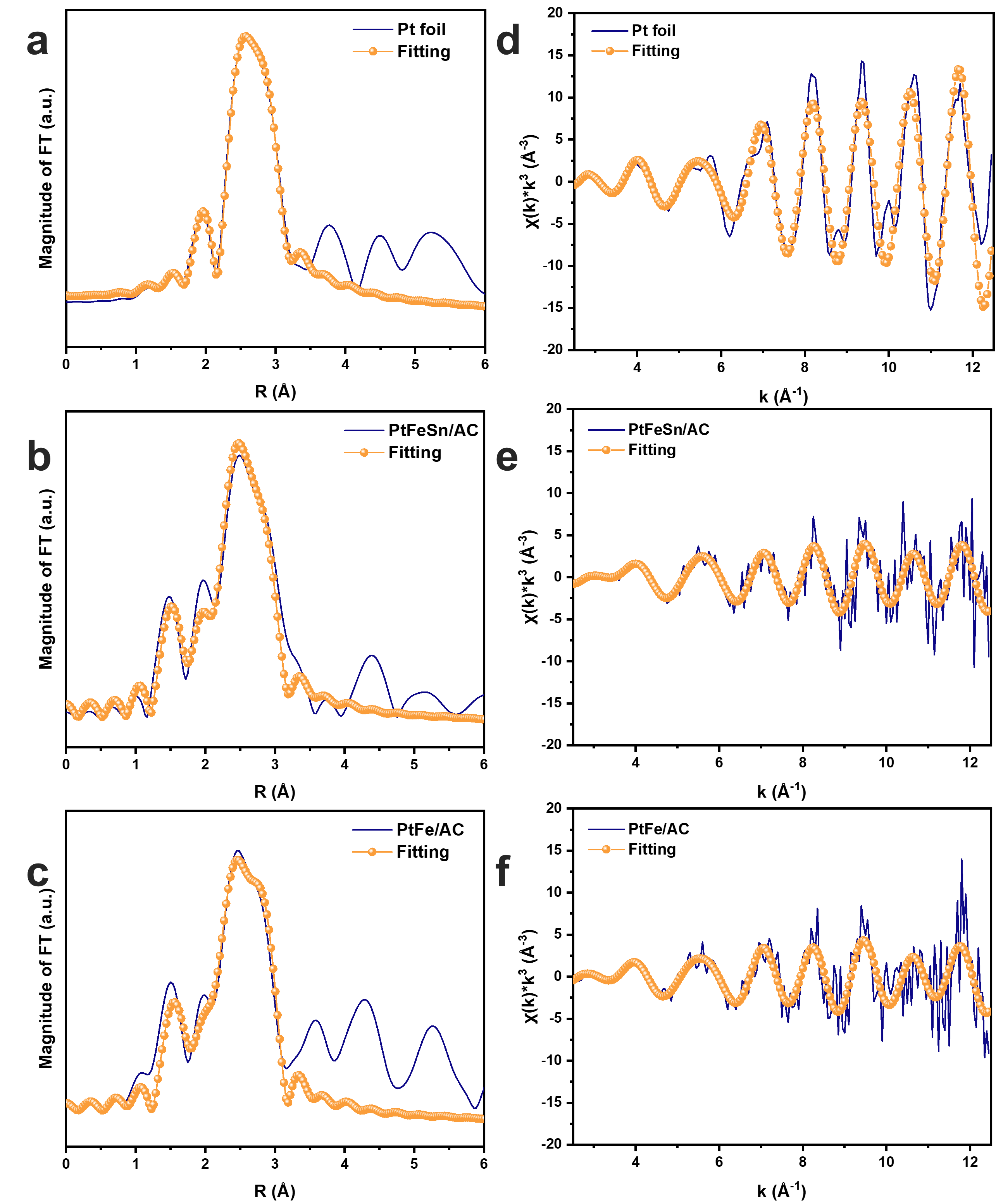


**Figure S9.** Fitted Fourier -Transformed R-space and k-space data of Pt L_3_-edge for Pt foil, PtFeSn/AC and PtFe/AC.


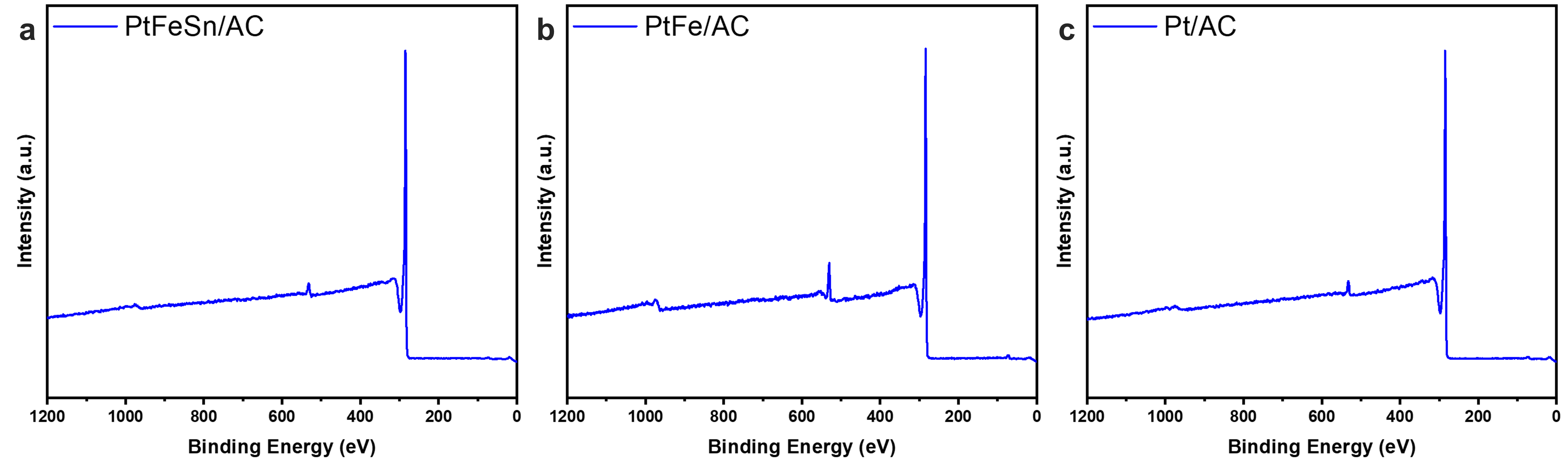


**Figure S10.** Characterizations of the electronic structures. Survey XPS spectra of a) PtFeSn/AC, b) PtFe/AC, c) Pt/AC.


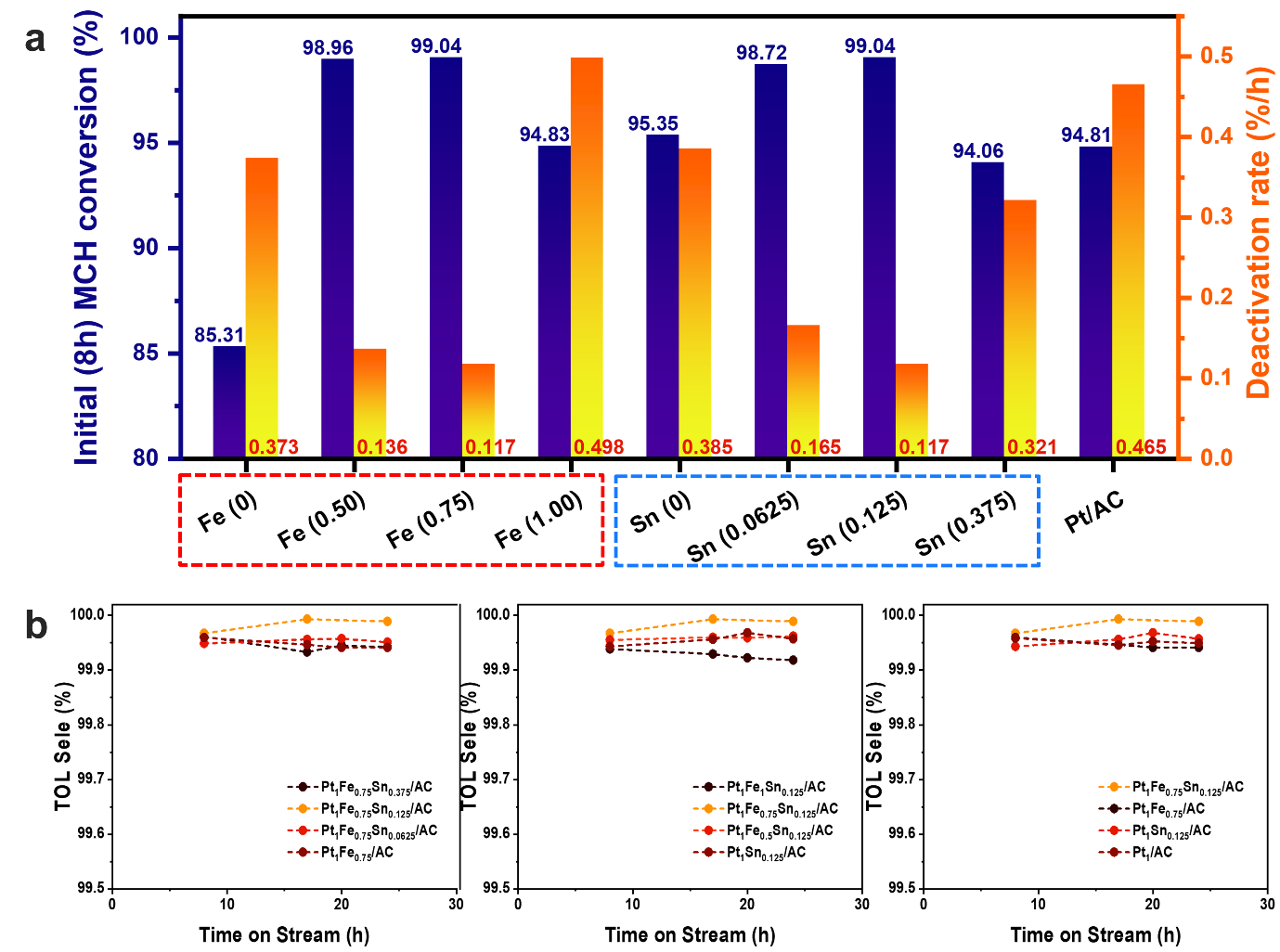


**Figure S11.** a) MCH conversion and deactivation rate of the LSL prepared catalysts with different Pt:Fe:Sn ratio (red-dash square: fix Pt:Sn ratio = 1:0.125, blue-dash square: fix Pt:Fe ratio = 1:0.75 (MCH flow rate at 25 ºC, 1.5 atm = 0.24 mL·min^-1^, weight of catalyst = 400 mg WHSV = 27.72 h^-1^, T = 375 ºC). b) the corresponding selectivity results.

To investigate the proper ratio of Pt-Fe-Sn, we fixed the Pt to Sn salt molar ratio to 1:0.125, while the Fe salt ratio was increased from 0 to 1, as shown in Figure S11a, (red squared). The initial MCH conversion is observed to be the highest, with lowest deactivation rate when the Fe salt ratio is 0.75. Subsequently, the Pt to Fe ratio is fixed as 1:0.75, and the Sn salt ratio was increased from 0 to 0.375 as shown in Figure S11a, Supporting Information (blue squared). The corresponding TOL selectivity results are presented in Figure S11b showing that all samples exhibit selectivity above 99.9%. When the Pt to Fe to Sn atomic ratio equals to 1:0.75:0.125, the catalyst (PtFeSn/AC) demonstrates the best dehydrogenation performance during the screening test.


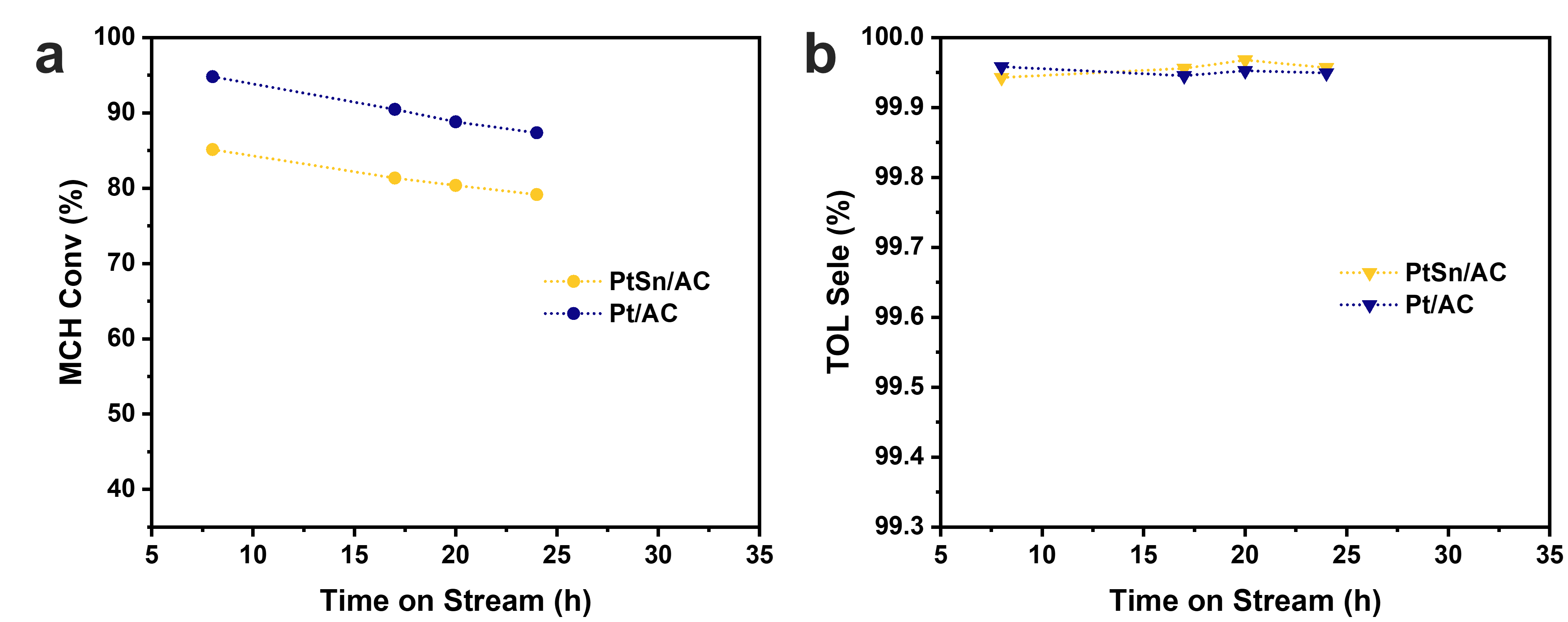


**Figure S12.** Evolution of the catalytic MCH dehydrogenation performance of the as-prepared catalysts. a, b) MCH conversion and TOL selectivity curves PtSn/AC, and Pt/AC (MCH flow rate at 25 ºC, 1.5 atm = 0.24 mL·min^-1^, weight of catalyst = 400 mg WHSV = 27.72 h^-1^, T = 375 ºC.


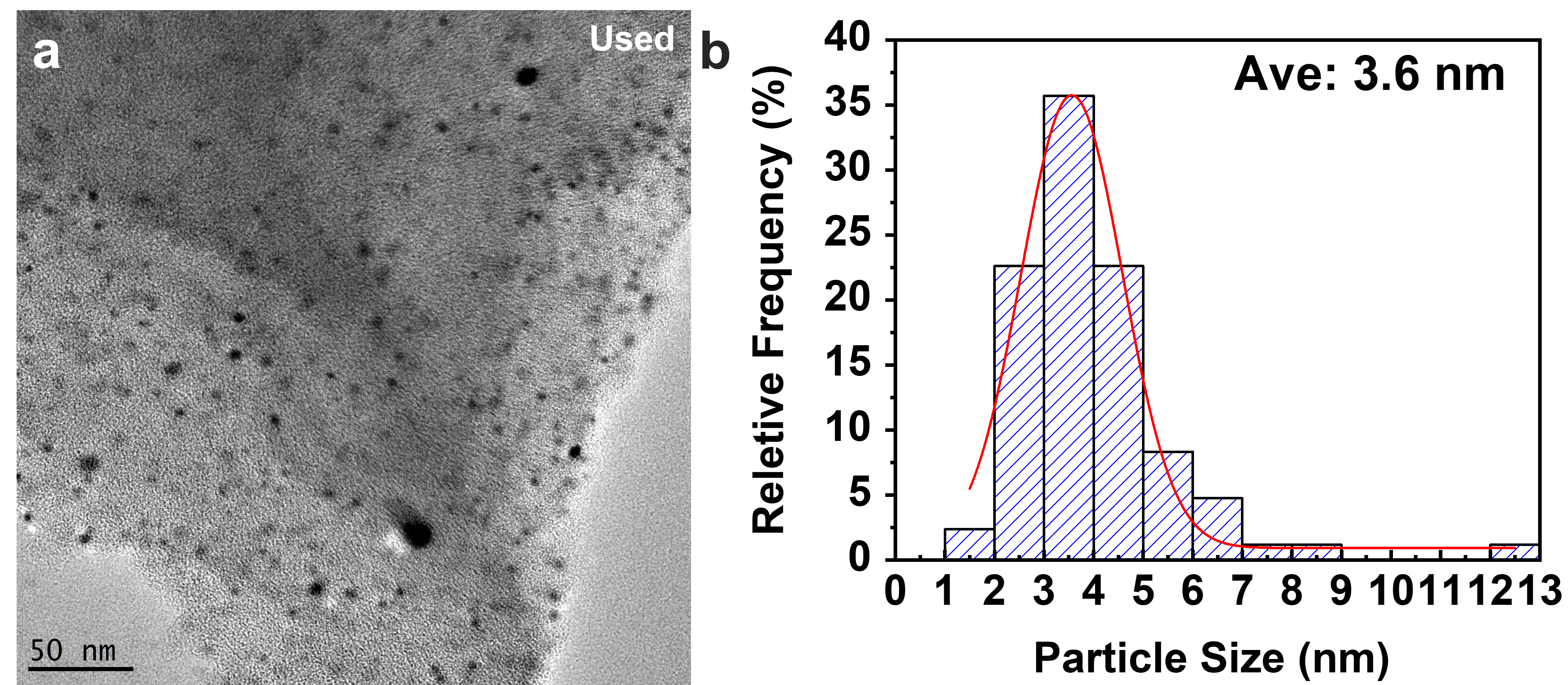


**Figure S13.** a) HRTEM image and b) The corresponding particle size distribution of used WI-PtFeSn/AC


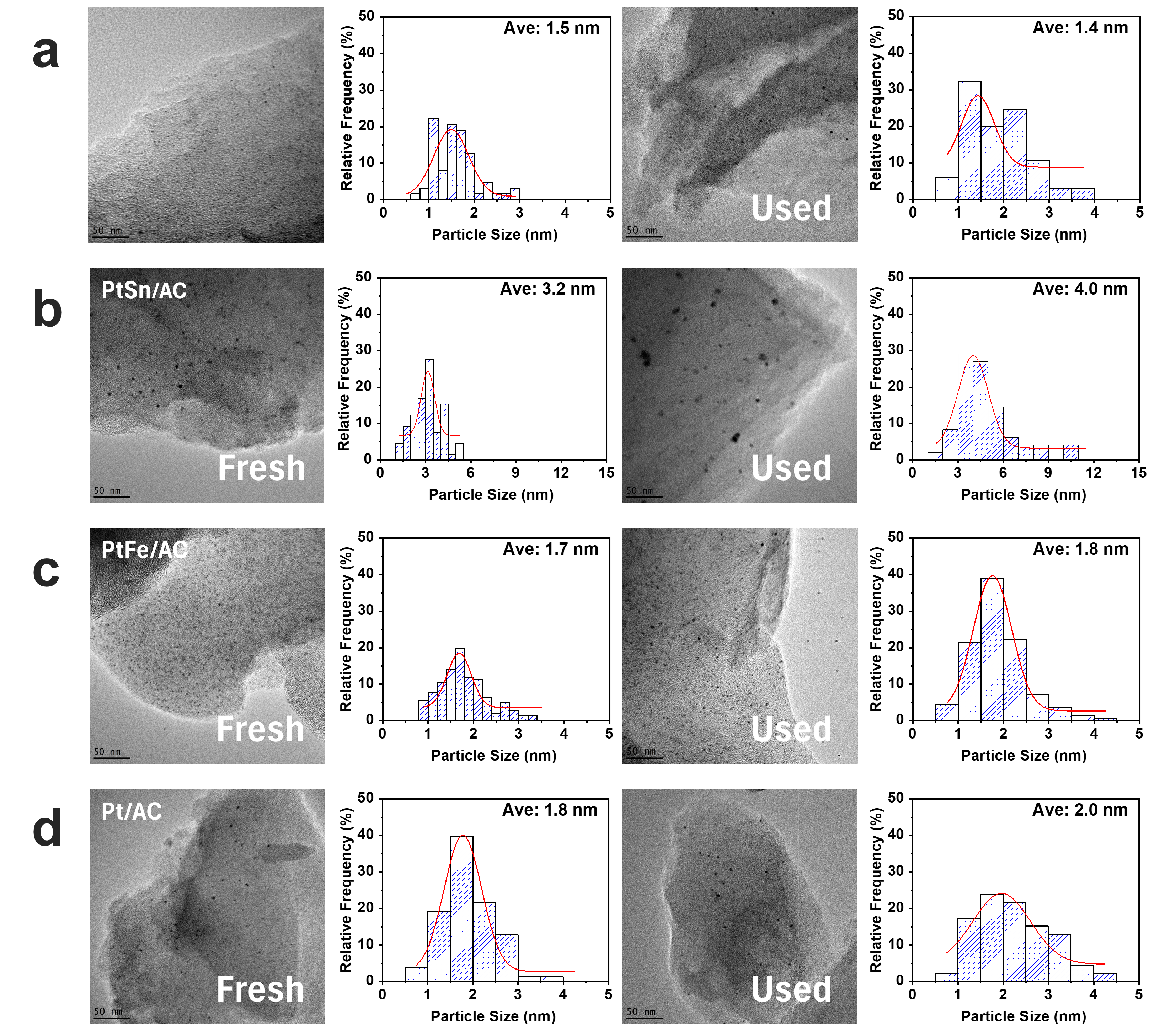


**Figure S14.** HRTEM images and the corresponding histograms of the statistics of the nanoparticle size distribution of a-d) fresh and used PtFeSn/AC, PtSn/AC, PtFe/AC, Pt/AC.

**
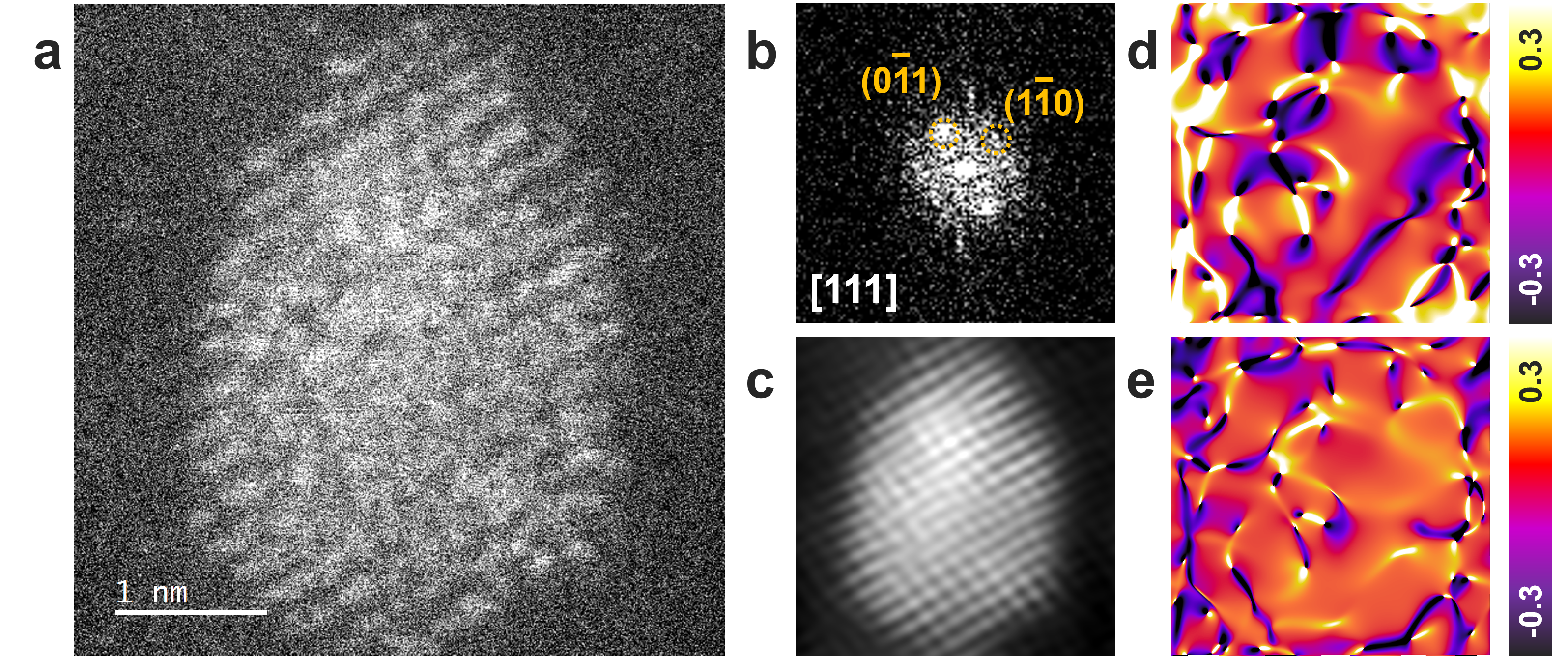
**

**Figure S15.** Microscopy and strain characterization of used PtFeSn/AC nanoparticle. a) HRTEM images of PtFeSn/AC nanoparticle. b) Corresponding Fast Fourier transfer (FFT) pattern of the nanoparticle. c) Inverse FFT (IFFT) patterns of b). d, e) The strain distributions in geometric phase image of Ɛxx and Ɛxy direction of the nanoparticle.


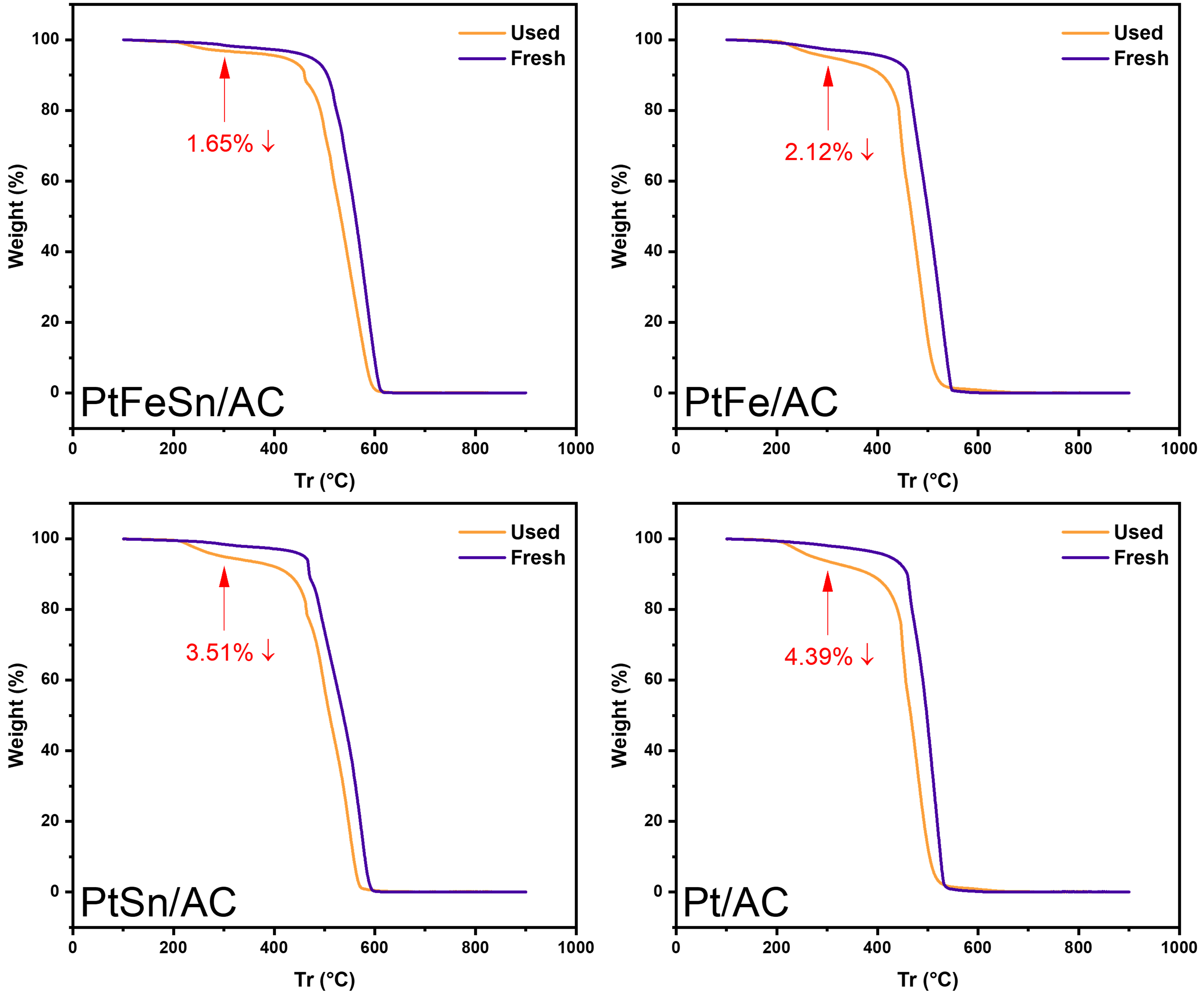


**Figure S16.** Thermalgravimetric analysis of a-d) PtFeSn/AC, PtFe/AC, PtSn/AC, and Pt/AC in air.


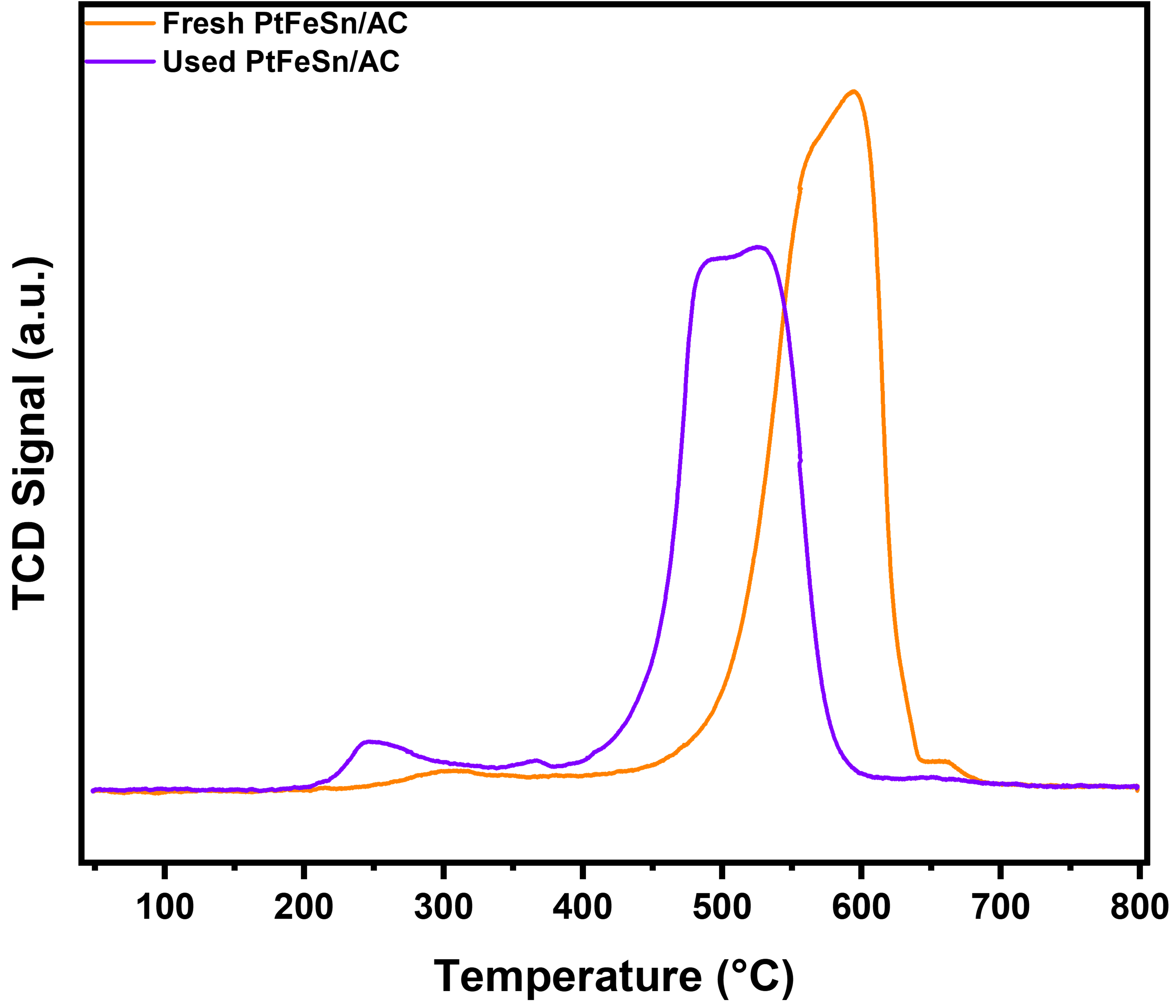


**Figure S17.** Temperature programmed oxidation analysis of fresh and used PtFeSn/AC.

**
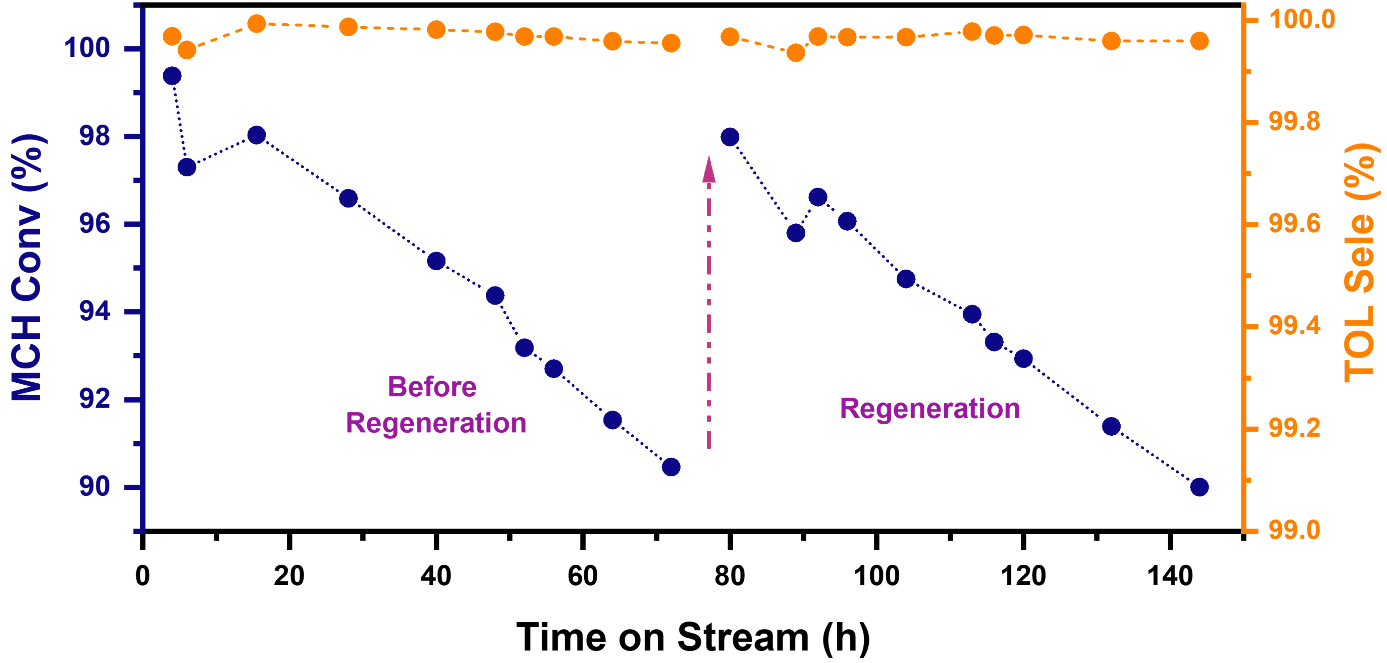
**

**Figure S18.** MCH conversion and TOL selectivity results before and after regeneration, the catalyst is regenerated by a flowing of air at 300 ^o^C for 1 h (200 mL·min^-1^) followed by H_2_ regeneration at 400 ^o^C for 2 h (200 mL·min^-1^), MCH flow rate at 25 ºC, 1.5 atm = 0.24 mL·min^-1^, weight of catalyst = 400 mg WHSV = 27.7 h^-1^, T = 375 ºC.


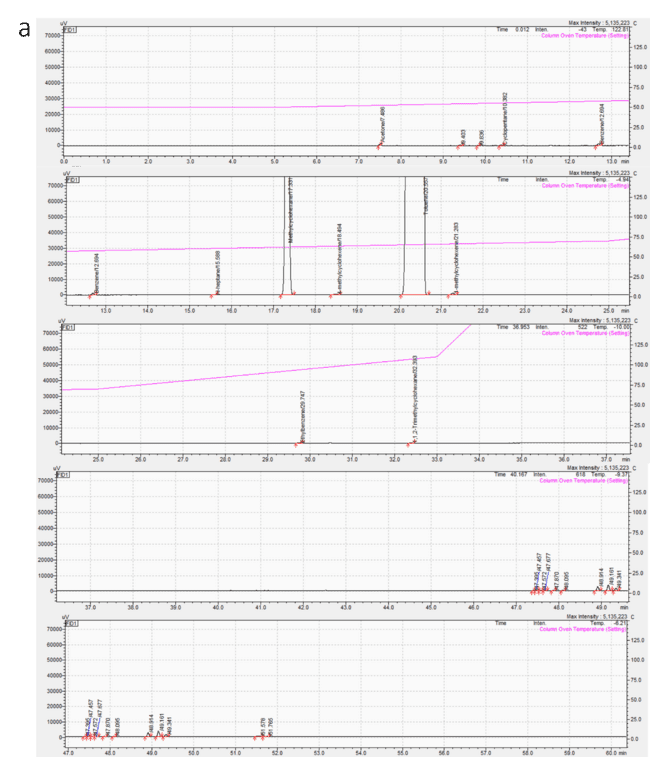

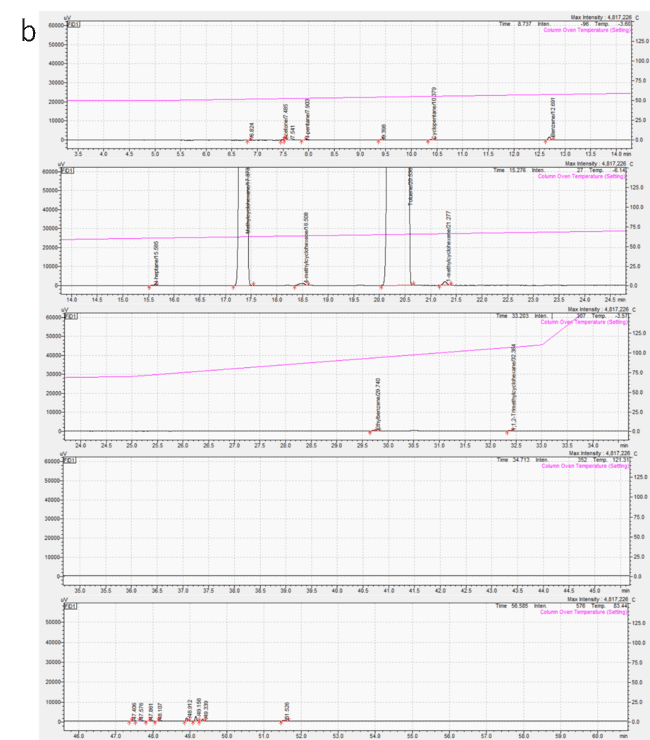


**Figure S19.** Gas chromatography (GC) curves of the liquid phase products over (a) PtFeSn/AC (24 h time-on-stream) and (b) Pt/AC (24 h time-on-stream). The pink line representing the oven temperature program.


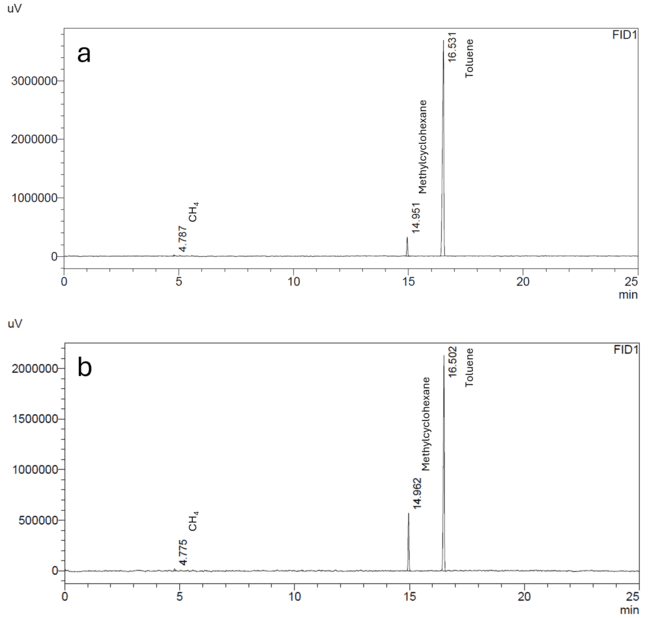


Figure S20. Gas chromatography (GC) curves of the gas phase products over a) PtFeSn/AC (24 h time-on-stream) and (b) Pt/AC (24 h time-on-stream).

| **Sample** | **Theoretical Pt wt%** | **Theoretical Fe wt%** | **Theoretical Sn wt%** | **Pt wt%** | **Fe wt%** | **Sn wt%** |
| --- | --- | --- | --- | --- | --- | --- |
| Pt/AC | 1 | \ | \ | 0.43 | \ | \ |
| PtSn/AC | 1 | \ | 0.12 | 0.59 | \ | 0.05 |
| PtFe/AC | 1 | 0.25 | \ | 0.64 | 0.18 | \ |
| PtFeSn/AC | 1 | 0.25 | 0.12 | 0.52 | 0.14 | 0.04 |
| WI-PtFeSn/AC | 1 | 0.25 | 0.12 | 0.55 | 0.20 | 0.02 |

**Table S1.** ICP-OES analysis. Corresponding metal weight percentage of Pt/AC, PtSn/AC, PtFe/AC, PtFeSn/AC and WI-PtFeSn/AC.

**Table S2.** Structural parameters of samples extracted from Pt L_3_-edge and Fe K edge EXAFS fitting.

| **Samples** | **Path** | **C.N.** | **R (Å)** | **σ^2^ (10^-3^ Å^2^)** | **ΔE_0_ (eV)** | **R factor** |
| --- | --- | --- | --- | --- | --- | --- |
| Pt-Foil | Pt-Pt | 12 | 2.76 ± 0.03 | 3.89 | 7.6 | 0.005 |
| PtFeSn/AC | Pt-O | 1.1 ± 0.2 | 1.97 ± 0.04 | 4.21 | 7.7 | 0.019 |
|  | Pt-Pt | 4.6 ± 0.6 | 2.73 ± 0.06 | 6.00 |  |  |
|  | Pt-M | 2.2 ± 0.5 | 2.66 ± 0.08 | 9.95 |  |  |
| PtFe/AC | Pt-O | 1.0 | 1.98 ± 0.04 | 1.43 | 5.3 | 0.018 |
|  | Pt-Pt | 6.7 ± 1.1 | 2.74 ± 0.01 | 8.00 |  |  |
|  | Pt-M | 3.0 | 2.70 ± 0.09 | 7.24 |  |  |

The cure fitting was conducted in R-space R-factor of fit indicated in the table, where C.N. is the coordination number, R is the distance between absorber and backscatter atoms, σ^2^ is the Debye-Waller factor value, and R-factor is characterizing the goodness of fitting.

S_0_^2^ is obtained by fitting Pt and Fe foil: 0.73 for Pt.

**Table S3.** Comparison between this work and academic benchmarks.

| **No** | **Catalyst** | **Pt loading (wt%)** | **Initial MCH conversion (%)** | **Final MCH**  **conversion (%)** | **T (ºC)** | **Max. rxn. time (h)** | **H_2_ evolution rate (mmol·g _Pt_^-1^·min^-1^)** | **WHSV (h^-1^)** |  | **Reference** |
| --- | --- | --- | --- | --- | --- | --- | --- | --- | --- | --- |
| 1 | PtFeSn/AC | 0.5 | 98.9 | 95.7 | 375 | 193 | 1313 | 13.9 |  | This work |
| 2 | PtFeSn/AC | 0.5 | 99.0 | 96.2 | 375 | 32 | 2625 | 27.8 |  | This work |
| 3 | Pt/Mg-Al-O | 0.5 | 99.0 | 92.0 | 350 | 218 | 1892 | 9.2 |  | ^[1]^ |
| 4 | Pt/Ce-Mg-Al-O | 0.4 | 98.5 | stable | 350 | 10 | 1358 | 9.2 |  | ^[2]^ |
| 5 | Pt-B/Al_2_O_3_ | 1.0 | 81.5 | 81.5 | 350 | 10 | 961 | 23.1 |  | ^[3]^ |
| 6 | Pt-Fe-Zn/SiO_2_ | 3.0 | 71.2 | - | 350 | - | 757 | - |  | ^[4]^ |
| 7 | Pt-Cu/Al_2_O_3_ | 1.4 | 92.6 | stable | 400 | 10 | 445 | 4.6 |  | ^[5]^ |
| 8 | Pt-Mn/Al_2_O_3_ | 1.0 | 89.0 | 85.0 | 350 | 3 | 369 | 27.7 |  | ^[6]^ |
| 9 | Pt/TiO_2_ | 1.0 | 60.0 | 60.0 | 350 | 25 | 249 | - |  | ^[7]^ |
| 10 | Pt/TiO_2_-Al_2_O_3_ | 0.5 | 95.0 | 50.0 | 400 | 1.5 | 146 | 4.6 |  | ^[8]^ |
| 11 | Pt/AC | 1.0 | 90.0 | 68.0 | 300 | 30 | 121 | - |  | ^[9]^ |

WHSV was not reported in some studies; therefore, it was calculated where sufficient data were available. For the remaining works, WHSV could not be determined due to missing information on MCH flow rate and/or catalyst weight.

**Table S4.** XPS fitting parameters of Pt 4f spectra for Pt/AC, PtFe/AC, and PtFeSn/AC catalysts. (BE: Binding Energy. FWHM: Full Width at Half Maximum)

| **Sample Name** | **Component** | **BE (eV)** | **FWHM (eV)** | **Spin–orbit Δ (eV)** | **Area ratio (4f₇⁄₂:4f₅⁄₂)** |
| --- | --- | --- | --- | --- | --- |
| PtFeSn/AC | Pt (metal) | 71.8 | 1.0 | 3.35 (± 0.1) | 1.3 |
|  | PtO | 72.8 | 1.2 | 3.35 (± 0.1) | 1.3 |
| PtFe/AC | Pt (metal) | 71.7 | 1.0 | 3.35 (± 0.1) | 1.3 |
|  | PtO | 72.7 | 1.1 | 3.35 (± 0.1) | 1.3 |
| Pt/AC | Pt (metal) | 71.6 | 1.0 | 3.35 (± 0.1) | 1.3 |
|  | PtO | 72.7 | 1.2 | 3.35 (± 0.1) | 1.3 |

**Table S5.** GC-FID analysis of liquid-phase products from the dehydrogenation of methylcyclohexane over Pt/AC, PtFe/AC, and PtFeSn/AC catalysts. All reactions were carried out at 375 °C under a WHSV of 27.7 h⁻¹.

| **Catalyst** | **MCH Conversion (%)** | **TOL selectivity (%)** | **Selectivity to side products** | | | | |
| --- | --- | --- | --- | --- | --- | --- | --- |
|  |  |  | **5-membered rings (%)** | **6-membered rings (%)** | **Benzene (%)** | **Paraffins (%)** | **Dimers (%)** |
| PtFeSn/AC  (24 h time-on-stream) | 97.2 | 99.9557 | 0.0014 | 0.0188 | 0.0055 | 0.0013 | 0.0173 |
| Pt/AC  (24 h time-on-stream) | 87.4 | 99.9331 | 0.0026 | 0.0353 | 0.0094 | 0.0028 | 0.0168 |

**Reference**

[1] Wu, K.,Chen, F.,Wang, F.,Huang, Y.,Shen, Z.,Wang, W.,Yang, Y. Preparation of Pt Supported on Mesoporous Mg–Al Oxide Catalysts for Efficient Dehydrogenation of Methylcyclohexane. *International Journal of Hydrogen Energy* **2021**, *46* (50), 25513–25519.

[2] Wang, W.,Miao, L.,Wu, K.,Chen, G.,Huang, Y.,Yang, Y. Hydrogen Evolution in the Dehydrogenation of Methylcyclohexane over Pt/Ce Mg Al O Catalysts Derived from Their Layered Double Hydroxides. *International Journal of Hydrogen Energy* **2019**, *44* (5), 2918–2925.

[3] Wu, X.,Lu, H.,Xiao, Y.,Guo, H.,Jia, L.,Li, D. Acid Site Introduced by Al3+penta and Boron in Pt/Al2O3 Catalyst for Dehydrogenation of Methylcyclohexane. *International Journal of Hydrogen Energy* **2022**, *47* (82), 34955–34962.

[4] Nakaya, Y.,Miyazaki, M.,Yamazoe, S.,Shimizu, K.,Furukawa, S. Active, Selective, and Durable Catalyst for Alkane Dehydrogenation Based on a Well-Designed Trimetallic Alloy. *ACS Catal.* **2020**, *10* (9), 5163–5172.

[5] Zhang, X.,He, N.,Lin, L.,Zhu, Q.,Wang, G.,Guo, H. Study of the Carbon Cycle of a Hydrogen Supply System over a Supported Pt Catalyst: Methylcyclohexane–Toluene–Hydrogen Cycle. *Catal. Sci. Technol.* **2020**, *10* (4), 1171–1181.

[6] Nakano, A.,Manabe, S.,Higo, T.,Seki, H.,Nagatake, S.,Yabe, T.,Ogo, S.,Nagatsuka, T.,Sugiura, Y.,Iki, H.,Sekine, Y. Effects of Mn Addition on Dehydrogenation of Methylcyclohexane over Pt/Al2O3 Catalyst. *Applied Catalysis A: General* **2017**, *543*, 75–81.

[7] Nagatake, S.,Higo, T.,Ogo, S.,Sugiura, Y.,Watanabe, R.,Fukuhara, C.,Sekine, Y. Dehydrogenation of Methylcyclohexane over Pt/TiO2 Catalyst. *Catal Lett* **2016**, *146* (1), 54–60.

[8] Yang, X.,Song, Y.,Cao, T.,Wang, L.,Song, H.,Lin, W. The Double Tuning Effect of TiO2 on Pt Catalyzed Dehydrogenation of Methylcyclohexane. *Molecular Catalysis* **2020**, *492*, 110971.

[9] Li, X.,Ma, D.,Xinhe, B. Dispersion of Pt Catalysts Supported on Activated Carbon and Their Catalytic Performance in Methylcyclohexane Dehydrogenation. *Chinese Journal of Catalysis* **2008**, *29* (3), 259–263.
